# APOBEC3D and APOBEC3F Potently Promote HIV-1 Diversification and Evolution in Humanized Mouse Model

**DOI:** 10.1371/journal.ppat.1004453

**Published:** 2014-10-16

**Authors:** Kei Sato, Junko S. Takeuchi, Naoko Misawa, Taisuke Izumi, Tomoko Kobayashi, Yuichi Kimura, Shingo Iwami, Akifumi Takaori-Kondo, Wei-Shau Hu, Kazuyuki Aihara, Mamoru Ito, Dong Sung An, Vinay K. Pathak, Yoshio Koyanagi

**Affiliations:** 1 Laboratory of Viral Pathogenesis, Institute for Virus Research, Kyoto University, Kyoto, Kyoto, Japan; 2 Viral Mutation Section, HIV Drug Resistance Program, Center for Cancer Research, National Cancer Institute-Frederick, Frederick, Maryland, United States of America; 3 Department of Biology, Faculty of Sciences, Kyushu University, Fukuoka, Fukuoka, Japan; 4 Department of Hematology and Oncology, Graduate School of Medicine, Kyoto University, Kyoto, Kyoto, Japan; 5 Viral Recombination Section, HIV Drug Resistance Program, Center for Cancer Research, National Cancer Institute-Frederick, Frederick, Maryland, United States of America; 6 Institute of Industrial Science, The University of Tokyo, Meguro-ku, Tokyo, Japan; 7 Graduate School of Information Science and Technology, The University of Tokyo, Meguro-ku, Tokyo, Japan; 8 Central Institute for Experimental Animals, Kawasaki, Kanagawa, Japan; 9 Division of Hematology and Oncology, University of California, Los Angeles, Los Angeles, California, United States of America; 10 School of Nursing, University of California, Los Angeles, Los Angeles, California, United States of America; 11 AIDS Institute, University of California, Los Angeles, Los Angeles, California, United States of America; University of Pennsylvania School of Medicine, United States of America

## Abstract

Several APOBEC3 proteins, particularly APOBEC3D, APOBEC3F, and APOBEC3G, induce G-to-A hypermutations in HIV-1 genome, and abrogate viral replication in experimental systems, but their relative contributions to controlling viral replication and viral genetic variation *in vivo* have not been elucidated. On the other hand, an HIV-1-encoded protein, Vif, can degrade these APOBEC3 proteins via a ubiquitin/proteasome pathway. Although APOBEC3 proteins have been widely considered as potent restriction factors against HIV-1, it remains unclear which endogenous APOBEC3 protein(s) affect HIV-1 propagation *in vivo*. Here we use a humanized mouse model and HIV-1 with mutations in Vif motifs that are responsible for specific APOBEC3 interactions, DRMR/AAAA (4A) or YRHHY/AAAAA (5A), and demonstrate that endogenous APOBEC3D/F and APOBEC3G exert strong anti-HIV-1 activity *in vivo*. We also show that the growth kinetics of 4A HIV-1 negatively correlated with the expression level of APOBEC3F. Moreover, single genome sequencing analyses of viral RNA in plasma of infected mice reveal that 4A HIV-1 is specifically and significantly diversified. Furthermore, a mutated virus that is capable of using both CCR5 and CXCR4 as entry coreceptor is specifically detected in 4A HIV-1-infected mice. Taken together, our results demonstrate that APOBEC3D/F and APOBEC3G fundamentally work as restriction factors against HIV-1 *in vivo*, but at the same time, that APOBEC3D and APOBEC3F are capable of promoting viral diversification and evolution *in vivo*.

## Author Summary

Mutation can produce three outcomes in viruses: detrimental, neutral, or beneficial. The first one leads to abrogation of virus replication because of error catastrophe, while the last one lets the virus escape from anti-viral immune system or adapt to the host. Human APOBEC3D, APOBEC3F, and APOBEC3G are cellular cytidine deaminases which cause G-to-A mutations in HIV-1 genome. Here we use a humanized mouse model and demonstrate that endogenous APOBEC3F and APOBEC3G induce G-to-A hypermutation in viral genomes and exert strong anti-HIV-1 activity *in vivo*. We also reveal that endogenous APOBEC3D and/or APOBEC3F induce viral diversification, which can lead to the emergence of a mutated virus that converts its coreceptor usage. Our results suggest that APOBEC3D and APOBEC3F are capable of promoting viral diversification and functional evolution *in vivo*.

## Introduction

Activation-induced cytidine deaminase/apolipoprotein B mRNA editing enzyme, catalytic polypeptide-like (AID/APOBEC) superfamily is composed of cellular cytidine deaminases that closely associate with crucial events in vertebrates such as immunity, malignancy, metabolism, and infectious diseases [1], [2]. For instance, AID causes somatic hypermutation in B cells resulting in antibody diversification [2], whereas APOBEC1 edits the mRNA of apolipoprotein B and regulates lipid metabolism [3].

The paralogs of human *AID*, *APOBEC1*, and *APOBEC2* genes are encoded in rodents and artiodactyls [4]. On the other hand, although mice encode a sole *Apobec3* gene, primates encode seven paralogs of murine *Apobec3* in their genome, which are designated to *APOBEC3A* to *H*. Given the strong evidence that the duplicated genes have been exposed to selective pressures [5], the seven *APOBEC3* genes have been positively selected [6] and APOBEC3 family proteins play various roles in primates including humans. For instance, APOBEC3A initiates the mutations of foreign DNA (e.g., microbial DNA), which leads to the clearance of bacteria from human cells [7]. In addition, APOBEC3B-mediated mutation closely associates with several human cancers [8], [9], particularly breast cancer [10].

APOBEC3G is the most extensively studied APOBEC3 protein in the field of virology and plays a crucial role in the infection and replication of HIV-1, a causative agent of AIDS [11]. APOBEC3G is incorporated into HIV-1 particles and induces G-to-A mutations in the newly synthesized viral DNA, which results in the abrogation of viral replication [4], [12]. On the other hand, an HIV-1-encoded protein, viral infectivity factor (Vif), impedes APOBEC3G incorporation into progeny virions by degrading these proteins through the ubiquitin/proteasome-dependent pathway [4], [12]. In addition to APOBEC3G, *in vitro* studies using cell culture systems have demonstrated that like APOBEC3G, APOBEC3F and APOBEC3D also potently impair HIV-1 replication [13]–[15]. However, one study has concluded that APOBEC3F expression levels in T cell lines were not sufficient to inhibit HIV-1 replication [16]. Another study analyzed the replication of HIV-1 Vif mutants that were defective in inducing degradation of APOBEC3G or APOBEC3F in primary CD4^+^ T cells, and concluded that APOBEC3G exerts a stronger antiviral activity on HIV-1 than APOBEC3F [17]. Thus, the relative impact of different APOBEC3 proteins on HIV-1 replication *in vivo* has not been determined.

Apart from their anti-HIV-1 abilities, certain studies have suggested that APOBEC3-mediated G-to-A mutation can lead to viral evolution and divergence [18]–[21]. However, it remains unclear how and which endogenous APOBEC3 proteins affect HIV-1 replication, pathogenesis, and diversity *in vivo*.

In order to elucidate the dynamics of HIV-1 infection *in vivo*, we have constructed a humanized mouse model by xenotransplanting human CD34^+^ hematopoietic stem cells (hHSCs) into an immunodeficient NOD/SCID *Il2rg^−/−^* (NOG) mouse [22]–[28]. Our humanized mouse model is able to recapitulate the characteristics of HIV-1 pathogenesis such as the depletion of peripheral CD4^+^ T cells [22], [23], [25]. By using this model, we have previously demonstrated that the expression levels of endogenous APOBEC3 genes in human CD4^+^ T cells of humanized mice were comparable to those of humans and that the combined activity of endogenous APOBEC3 proteins can potently abrogate *vif*-deficient HIV-1 propagation *in vivo* [23]. However, which endogenous APOBEC3 proteins are crucial to the anti-HIV-1 effect *in vivo* is not yet known. In fact, although G-to-A mutations, presumably caused by endogenous APOBEC3 proteins, have been clearly observed in the viral genomes of HIV-1-infected patients, the frequencies of G-to-A mutations seem to vary among individuals and the mutation context is still controversial [21], [29]–[37]. Moreover, because there is a possibility that some endogenous APOBEC3 protein(s) are capable of facilitating viral diversification *in vivo*, it is important to elucidate how endogenous APOBEC3 proteins would affect HIV-1 if we target these molecules for therapy.

In this study, we demonstrate that the propagation of HIV-1 *vif* mutants, which are unable to degrade APOBEC3D/F, APOBEC3G, or both APOBEC3D/F and APOBEC3G, are severely impaired, demonstrating that endogenous APOBEC3D/F and APOBEC3G proteins potently suppress HIV-1 propagation *in vivo*. In addition to the anti-HIV-1 activity of APOBEC3D and APOBEC3F, our results demonstrate that endogenous APOBEC3D and APOBEC3F also potently induced viral diversification. Taken together, our findings show APOBEC3D and APOBEC3F promote HIV-1 diversification *in vivo* and thereby facilitate viral adaptation and evolution.

## Results

### Strong inhibition of HIV-1 propagation *in vivo* by mutating ^14^DRMR^17^ and/or ^40^YRHHY^44^ motifs in Vif

**Figure 1:**
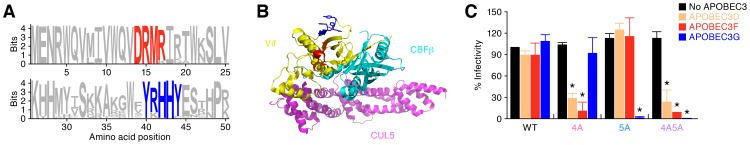
Anti-HIV-1 effect of APOBEC3 proteins *in vitro*. (A) Conservation of ^14^DRMR^17^ and ^40^YRHHY^44^ motifs in Vif. The *vif* ORF sequences of HIV-1 group M that are registered in Los Alamos HIV sequence database (n = 7,118) were aligned and analyzed as described in Materials and Methods. (B) Location of DRMR and YRHHY motifs in Vif crystal structure. The 3D structure of Vif was generated on PyMOL v1.6 (http://www.pymol.org/) with the crystal structure of Vif-CBFβ-CUL5-ELOB-ELOC complex (PDB code: 4N9F) [79]. Yellow, cyan, and magenta cartoons respectively represent the main chain of Vif, CBFβ, and CUL5. Red and blue cartoons respectively represent DRMR and YRHHY motifs in Vif. (C) TZM-bl assay. The infectivity of released virions was determined by using TZM-bl cells. The infectivity of each virus is normalized to the value of WT HIV-1 without APOBEC3. The assay was performed in triplicate. *P<0.05 versus no APOBEC3 by Student's *t* test. The assay was performed in triplicate. The data represents average with SD.

It was demonstrated that ^14^DRMR^17^ motif in Vif is necessary for the degradation of APOBEC3D and APOBEC3F, while ^40^YRHHY^44^ motif in Vif is necessary for the degradation of APOBEC3G [38], [39]. As shown in Figure 1A, these two motifs were highly conserved in HIV-1 group M. In addition, these motifs are located on the outside regions of Vif protein (Figure 1B) [39]. Moreover, we confirmed that APOBEC3D, APOBEC3F, and APOBEC3G have the ability to decrease *vif*-deficient HIV-1 infectivity *in vitro* (Figure S1).

To confirm the importance of these motifs *in vivo*, we prepared 3 Vif mutants, DRMR/AAAA (4A), YRHHY/AAAAA (5A), and a double mutant (4A5A), based on a CCR5-tropic HIV-1 infectious molecular clone (IMC; strain NLCSFV3) [40]. As previously reported [17], the infectivity of WT, 4A, 5A, and 4A5A HIV-1s were comparable in the absence of APOBEC3s (Figure 1C). On the other hand, the infectivity of 4A HIV-1 was strongly suppressed by APOBEC3D and APOBEC3F but not by APOBEC3G, while that of 5A HIV-1 was decreased by APOBEC3G but not by APOBEC3D and APOBEC3F (Figure 1C). These results indicate that 4A HIV-1 is sensitive to APOBEC3D and APOBEC3F but not to APOBEC3G, while 5A HIV-1 is sensitive to APOBEC3G but not to APOBEC3D and APOBEC3F.

**Figure 2:**
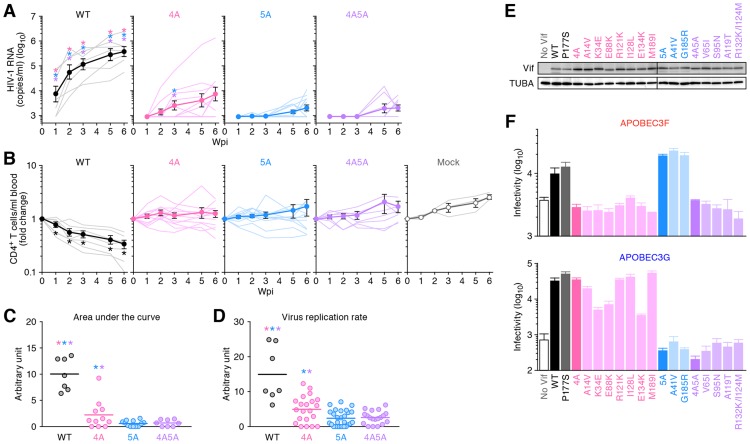
Dynamics of HIV-1 *vif* mutant infection in humanized mice. (A and B) The virus solutions containing 5 ng of p24 antigen (WT HIV-1 [n = 7], 4A HIV-1 [n = 11], 5A HIV-1 [n = 12], and 4A5A HIV-1 [n = 8]) or RPMI 1640 (n = 3; for mock infection) were inoculated into humanized mice, and the amount of viral RNA in plasma (A) and the level of peripheral CD4^+^ T cells (CD45^+^ CD3^+^ CD4^+^ cells) (B) were analyzed at 0, 1, 2, 3, 5, and 6 wpi. The averages are shown in circles with SEMs, and the values from each mouse are shown by line. X-axes, wpi. In panel A, the detection limit of HIV-1 RNA is 800 copies/ml plasma. (C) Area under the curve (AUC). AUCs of the VL of the mice infected with WT HIV-1 (n = 7), 4A HIV-1 (n = 11), 5A HIV-1 (n = 12), 4A5A HIV-1 (n = 8) were calculated using the trapezoidal rule as described in Materials and Methods. (D) Virus replication rate. Virus replication rates of WT HIV-1 (n = 7), 4A HIV-1 (n = 21), 5A HIV-1 (n = 27), and 4A5A HIV-1 (n = 19) were estimated by using the data of VL and peripheral CD4^+^ T cell counts as described in Materials and Methods. In panels C and D, horizontal bars represent the averages. Asterisks represent statistically significant differences (*P*<0.05 by Student's *t* test) versus each HIV-1 *vif* mutant (A), between WT HIV-1 and *vif* mutants (C and D), and between infected mice and mock-infected mice (B). In panels A, C, and D, each color of asterisk represents the statistically significant difference against each HIV-1 *vif* mutant-infected mice. (E and F) No Vif reversion in HIV-1 *vif* mutant-infected humanized mice. (E) Western blotting of the Vif mutants frequently observed in infected mice (see also Figure S3). The input of cell lysate was standardized to α-Tubulin (TUBA), and representative results are shown. (F) TZM-bl assay. The expression plasmids of the Vif mutants were cotransfected with pNLCSFV3*Δvif* and either APOBEC3F (*top*) or APOBEC3G (*bottom*) expression plasmids into 293T cells, and the infectivity of released virus was determined by using TZM-bl cells. The assay was performed in triplicate. The data represents average with SD.

To investigate the anti-HIV-1 activity of each endogenous A3 protein *in vivo*, we inoculated WT, 4A, 5A, and 4A5A HIV-1s into humanized mice. As shown in Figure 2A, the viral loads (VLs) of 4A, 5A, and 4A5A HIV-1s were significantly lower than that of WT HIV-1, and the gradual decrease of peripheral CD4^+^ T cells was observed only in WT HIV-1-infected mice (Figure 2B). In contrast to *vif*-deleted viruses that did not replicate at all in humanized mouse models [23], [41], 4A, 5A and 4A5A HIV-1s exhibited partial viremia (VL at 6 weeks postinfection [wpi]: WT HIV-1, 6.3×10^5^±3.0×10^5^ copies/ml; 4A HIV-1, 1.2×10^5^±1.2×10^5^ copies/ml; 5A HIV-1, 2.9×10^3^±0.9×10^3^ copies/ml; 4A5A HIV-1 2.3×10^3^±0.7×10^3^ copies/ml). These suggest that the *vif*-mutated viruses used in this study retain some Vif activity, even though they are highly defective. We also inoculated higher doses of viruses into humanized mice; however, despite the higher virus dose, these HIV-1 *vif* mutants did not propagate efficiently *in vivo* (Figures S2A and S2B). These findings strongly suggest that endogenous APOBEC3 proteins, particularly APOBEC3D, APOBEC3F and APOBEC3G, can potently impair HIV-1 propagation *in vivo*.

To quantitatively analyze the magnitude of viral propagation *in vivo*, we evaluated the area under the curve (AUC) of VL (Figure 2C; see also Materials and Methods) and the virus replication rate (Figure 2D; see also Materials and Methods). As shown in Figures 2C and 2D, these two analyses revealed that both the AUC and virus replication rate of WT HIV-1 were significantly higher than those of 4A, 5A, and 4A5A HIV-1s (AUC: WT HIV-1, 10.0±1.8; 4A HIV-1, 2.9±0.7; 5A HIV-1, 1.1±0.3; 4A5A HIV-1, 0.9±0.2. Virus replication rate: WT HIV-1, 15.0±3.0; 4A HIV-1, 4.9±0.8; 5A HIV-1, 2.4±0.4; 4A5A HIV-1, 2.6±0.4). In addition, although the differences in viral load (Figure 2A) and CD4 decline (Figure 2B) between 4A and 5A HIV-1-infected mice were not large, we detected statistically significant differences between 4A and 5A HIV-1-infected mice in AUC (3.8-fold, *P* = 0.030; Figure 2C) and virus replication rate (2.1-fold, *P* = 0.0050; Figure 2D), respectively. Taken together, these findings suggest that endogenous APOBEC3G, APOBEC3F and/or APOBEC3D have the potential to diminish HIV-1 propagation *in vivo* and that the antiviral activity of endogenous APOBEC3G is higher than the combined antiviral activity of APOBEC3D and APOBEC3F.

### No reversion of mutations in HIV-1 *vif* mutants

Although the growth of HIV-1 *vif* mutants was generally low, certain mice infected with 4A HIV-1 exhibited moderate levels of viremia (Figure 2A). To assess the possibility that reversion of mutations in *vif* led to the limited spread of HIV-1 *vif* mutants in humanized mice, we analyzed the *vif* mRNA sequences in the spleen of infected mice at 6 wpi. We observed prominent G-to-A mutations in HIV-1 *vif* mutant-infected mice (Figures S3A and S3B). We then asked whether nonsynonymous Vif mutants frequently identified in infected mice (Figure S3C) maintained their ability to degrade APOBEC3 proteins. As shown in Figure 2E, all Vif mutants were expressed at similar levels. However, although some minor variants in 4A HIV-1-infected mice such as K34E (3/320), E88K (5/320), and E134K (9/320) lost their-anti-APOBEC3G activity, all Vif mutants isolated from 4A HIV-1-infected mice were unable to eliminate APOBEC3F, and those from 5A HIV-1-infected mice were unable to eliminate APOBEC3G (Figure 2F). Although I128L mutant (*A*TA-to-*T*TA mutation) was predominantly observed in a 4A HIV-1-infected mouse (62 out of the 320 sequences analyzed; Figure S3C), this mutation did not affect its anti-APOBEC3 activity (Figure 2F). These results indicate that the *vif* mutations did not revert the 4A and 5A mutant phenotypes in infected mice, and that the moderate levels of viremia observed in some HIV-1 *vif* mutant-infected mice was not due to the restoration of Vif function.

### Negative correlation between the expression level of APOBEC3F and the growth of 4A HIV-1

**Figure 3:**
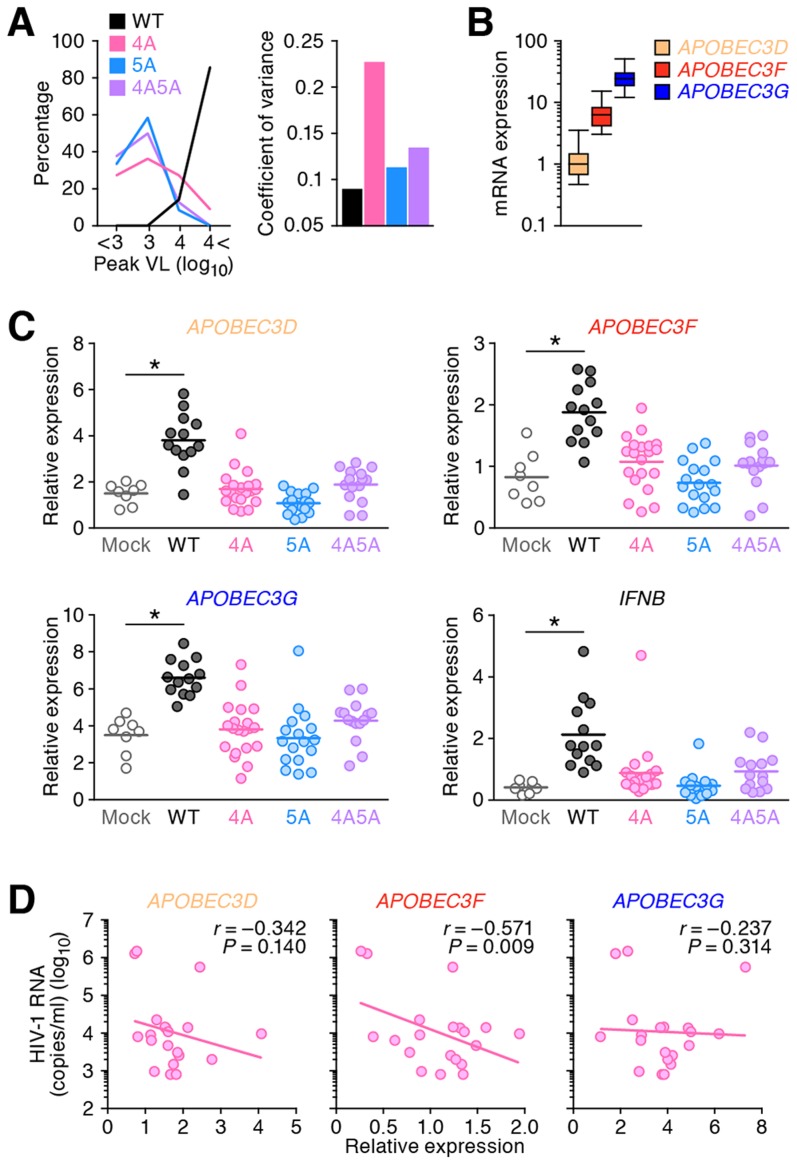
Expression levels of *APOBEC3* and *IFNB* in infected humanized mice. (A) Dispersion of HIV-1 growth efficiency in humanized mice. (*Left*) The peak VLs of WT HIV-1 (n = 7), 4A HIV-1 (n = 11), 5A HIV-1 (n = 12), and 4A5A HIV-1 (n = 8) were classified into 4 degrees (less than 10^3^, 10^3^–10^4^, 10^4^–10^5^, or more than 10^5^), and the distribution is plotted. (*Right*) The coefficient of variance of the peak VLs of WT HIV-1 (n = 7), 4A HIV-1 (n = 11), 5A HIV-1 (n = 12), and 4A5A HIV-1 (n = 8) is shown. (B) Expression levels of *APOBEC3D*, *APOBEC3F*, and *APOBEC3G* in the splenic human CD4^+^ T cells of humanized mice (n = 73) were analyzed by real-time RT-PCR. The values were standardized as previously described [43], and the level of *APOBEC3D* is set to 1 to facilitate comparison. (C) Expression levels of *APOBEC3D*, *APOBEC3F*, *APOBEC3G*, and *IFNB* in the splenic human CD4^+^ T cells of infected mice (WT, n = 13; 4A, n = 20; 5A, n = 17; and 4A5A, n = 15) and mock-infected mice (n = 8) at 6 wpi were analyzed by real-time RT-PCR. Horizontal bars represent the averages. Asterisks represent statistically significant difference (*P*<0.05 by Student's *t* test) between infected mice and mock-infected mice. (D) Negative correlation between VL and *APOBEC3* expression in 4A HIV-1-infected humanized mice. The mRNA expression levels of *APOBEC3D* (*left*), *APOBEC3F* (*middle*), and *APOBEC3G* (*right*) in the splenic human CD4^+^ T cells (x-axes) and the VL at 6 wpi (y-axis) of 4A HIV-1-infected mice (n = 20) are shown. The lines represent exponential approximation. Pearson correlation coefficient (*r*) was adopted to determine statistically significant correlation between each value.

In the 3 kinds of HIV-1 *vif* mutants, it was noteworthy that the VL in each 4A HIV-1-infected mouse varied between individual mice, while those in 5A and 4A5A HIV-1-infected mice were uniformly low (Figure 3A, left panel). In fact, the coefficient of variance of peak VL, which indicates the extent of distribution, in 4A HIV-1-infected mice was ∼2-fold higher than that in WT, 5A, and 4A5A HIV-1-infected mice (Figure 3A, right panel). These findings raised a possibility that the level of viremia in 4A HIV-1-infected mice is correlated with endogenous *APOBEC3* expression levels. To address this possibility, we determined the endogenous expression levels of *APOBEC3D, APOBEC3F*, and *APOBEC3G* in the spleen of humanized mice by real-time RT-PCR and standardized them to those of *APOBEC3D* according to the procedure reported previously [42], [43]. Since we have previously demonstrated that the spleen is one of the major tissues for HIV-1 replication in our hHSC-transplanted humanized mouse model [25], we assumed that the endogenous expression levels of *APOBEC3* genes in the splenic human mononuclear cells (MNCs) affect the kinetics of 4A HIV-1 growth. As shown in Figure 3B, the expression levels of *APOBEC3F* and *APOBEC3G* were 6.4-fold and 24.7-fold higher than those of *APOBEC3D*, respectively, and the expression level of *APOBEC3G* was 3.9-fold higher than that of *APOBEC3F*.

We then analyzed the endogenous expression level of each *APOBEC3* gene in infected mice. Consistent with a previous study in CD4^+^ T cell cultures *in vitro* [43], WT HIV-1 infection significantly enhanced the mRNA expression of *APOBEC3D*, *APOBEC3F*, and *APOBEC3G* (Figure 3C). Given that HIV-1 infection induces type I interferon (IFN) production in *in vitro* cell cultures [44] and infected individuals during the acute phase [45], taken together with the fact that type I IFNs potently enhance the expression of APOBEC3 genes [42], [46], we further evaluated the expression level of *IFNB*, a type I IFN. As shown in Figure 3C, the level of *IFNB* in WT HIV-1-infected mice is also significantly higher than that in mock-infected mice, although the levels of *APOBEC3D*, *APOBEC3F*, *APOBEC3G* and *IFNB* in HIV-1 *vif* mutant infected mice were comparable to mock-infected mice. Moreover, the expression levels of respective *APOBEC3* (Figure S4A) and *IFNB* (Figure S4B) were significantly correlated with each other. These findings suggest that HIV-1 propagation induces type I IFN production resulting in the augmentation of *APOBEC3* expression in humanized mice. In addition to type I IFNs, it has been reported that certain cytokines such as interleukin-2, 7, and 15 [47] and mitogens [43] also potently enhance *APOBEC3* expression. Therefore, the enhancement of *APOBEC3* expression observed in infected humanized mice (Figure 3C) might be a combination effect of type I IFNs and the other factors.

Furthermore, we assessed the relationship between the *APOBEC3* expression level and 4A HIV-1 growth kinetics and found that the VLs in 4A HIV-1-infected mice negatively correlated with the expression level of *APOBEC3F* but not of *APOBEC3D* with statistical significance (Figure 3D; *r* = −0.571, *P* = 0.009). Taken together, these results suggest that the endogenous expression level of *APOBEC3F* determines the growth kinetics of 4A HIV-1 *in vivo*.

### Distinct mutation signatures observed in HIV-1 *vif* mutant-infected humanized mice

**Figure 4:**
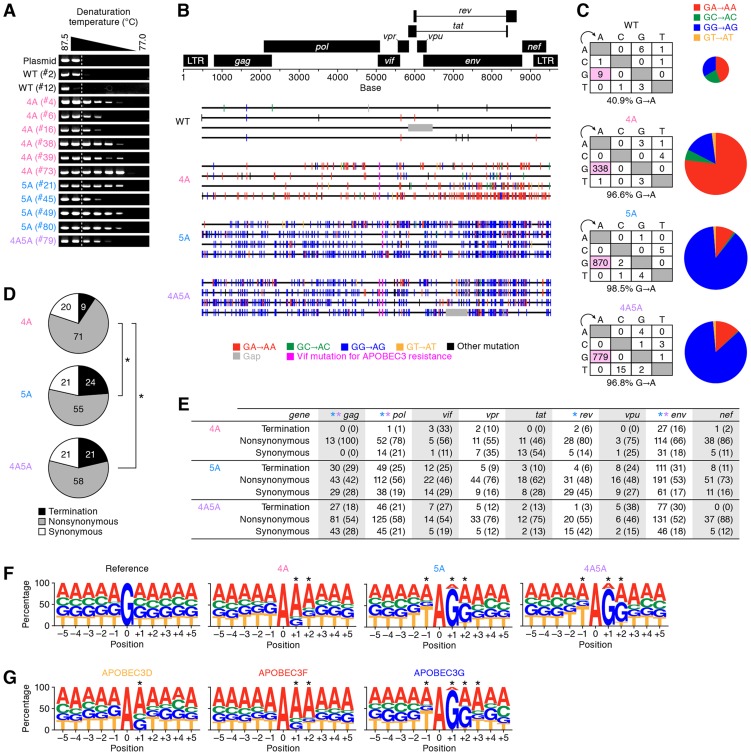
G-to-A hypermutation in the proviral DNA of infected humanized mice. (A) Semiquantitative 3D-PCR. Representative results are shown. Mouse IDs are shown in parentheses (correspond to those in Table S2). The dotted line indicates the lowest denaturation temperature (86.8°C) at which the PCR product is amplified from WT HIV-1-infected mice (mouse ID ^#^2). (B and C) Full-length proviral DNA were cloned and sequenced as described in Materials and Methods. Representative results (B), mutation matrix (C, *left*), and pie chart of G-to-A mutation (C, *right*) are respectively shown. The diameters of pie charts represent the percentage of G-to-A mutations in total mutations. (D and E) Effect of G-to-A mutation in proviral DNA. (D) Pie chart of the effect of G-to-A mutation in full-length proviral DNA. The numbers in pie chart represent the percentage of termination, nonsynonymous, and synonymous mutations in G-to-A mutations, respectively. (E) Summary of the effect of G-to-A mutation in proviral DNA. The numbers and the percentages (in parentheses) of termination, nonsynonymous, and synonymous G-to-A mutations in each viral gene are summarized. Asterisks represent statistically significant differences (*P*<0.01 by Chi-square test for independence). In panel E, each color of asterisk represents the statistically significant difference between 4A HIV-1 and each HIV-1 *vif* mutant. (F and G) G-to-A mutation sites in the proviral DNA of infected mice (F) and *in vitro* infection assay (G) were respectively classified according to the nucleotides positioned between −5 to +5 from the detected G-to-A mutation sites (position 0). The results were respectively compared to that expected if G-to-A mutations occurred randomly occurred (F, ‘reference’). *P<0.001 between the obtained and the expected results in each position by Chi-square test for independence. See also Figure S5.

To analyze the impact of APOBEC3-mediated mutations on the viral genome *in vivo*, we performed semiquantitative differential DNA denaturation PCR (3D-PCR) [48]. In this assay, if G-to-A mutations have accumulated in an amplicon, a PCR product can be detected even at lower denaturation temperatures because the decreased GC content in the amplicon leads to more efficient denaturation at lower temperatures [48], [49]. As shown in Figure 4A, the 3D-PCR products were detected at relatively lower denaturation temperatures in HIV-1 *vif* mutants but not of WT HIV-1, suggesting that the proviral genomes of HIV-1 *vif* mutants suffered from APOBEC3-mediated G-to-A hypermutation *in vivo*.

It is known that APOBEC3G predominantly generates *G*G-to-*A*G mutations, while APOBEC3D and APOBEC3F predominantly generate *G*A-to-*A*A mutations [14]. We assessed the sequence of full-length proviral DNA in the spleen of infected mice at 6 wpi, and found that *G*A-to-*A*A hypermutation was frequently observed in 4A HIV-1, while *G*G-to-*A*G hypermutation was readily observed in 5A and 4A5A HIV-1s (Figures 4B and 4C).

We then assessed the effect of G-to-A mutation detected in the proviral DNA of infected mice. As shown in Figure 4D, the results revealed that the percentage of termination codon mutations in 4A HIV-1 (9.3%) was significantly lower than those in 5A HIV-1 (23.8%) and 4A5A HIV-1 (21.3%) (4A HIV-1 versus 5A HIV-1, *P* = 0.62×10^−9^; 4A HIV-1 versus 4A5A HIV-1, *P* = 0.41×10^−6^ by Chi-square test for independence). Similar results were observed in the longer viral genes such as *gag*, *pol*, and *env* (Figure 4E). These results strongly suggest that APOBEC3G efficiently generates termination codons compared to APOBEC3D and APOBEC3F.

To investigate the trend of G-to-A mutation sites in depth, we verified the nucleotides positioned between −5 to +5 from the G-to-A mutation sites in the proviral DNA. Comparing the observed mutations to the expected random G-to-A mutations (shown as “reference” in Figure 4F), statistical analyses revealed that the mutation signature of 4A HIV-1-infected mice is *G*AA-to-*A*AA, while those of 5A and 4A5A HIV-1-infected mice were T*G*GG-to-T*A*GG (Figures 4F and S5A). Moreover, *in vitro* single-round infection assays revealed that the mutation signatures of APOBEC3D, APOBEC3F, and APOBEC3G were *G*A-to-*A*A, *G*AA-to-*A*AA, and T*G*GG-to-T*A*GG, respectively (Figure 4G). These results indicate that the mutation signature observed in 4A HIV-1-infected was statistically similar to those of APOBEC3D and/or APOBEC3F, and that those in 5A and 4A5A HIV-1-infected mice were statistically similar to that of APOBEC3G (Figure S5B).

### Diversification of 4A HIV-1 *in vivo*

**Figure 5:**
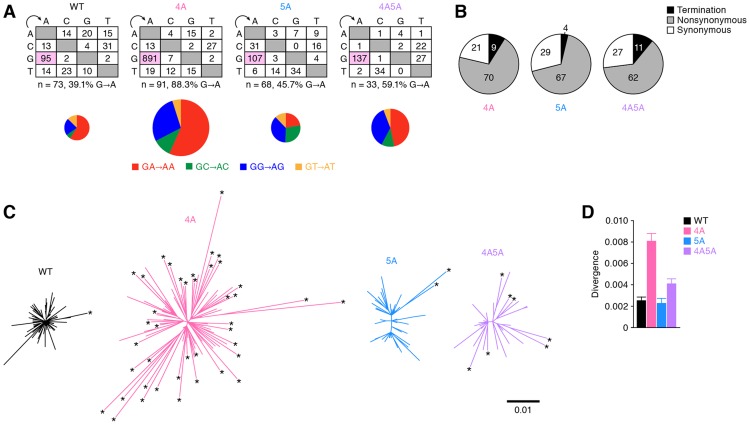
Diversification and functional evolution of 4A HIV-1 *in vivo*. The *env* ORFs (6221–8782, 2,562 bases) of viral RNA in the plasma of infected mice (WT, n = 73 from 2 mice; 4A, n = 91 from 3 mice; 5A, n = 68 from 2 mice; and 4A5A, n = 33 from 1 mouse) were sequenced by SGS assay. Raw data are shown in Figure S7. (A) The mutation matrix (*top*) and the pie chart of G-to-A mutation (*bottom*) are shown. In the bottom panel, the diameters of pie charts represent the percentage of G-to-A mutations in total mutations. (B) Effect of G-to-A mutation in *env* ORF of viral RNA in plasma. Pie chart of the effect of G-to-A mutation in *env* ORF is shown. The numbers in pie chart represent the percentage of termination, nonsynonymous, and synonymous mutations in G-to-A mutations, respectively. (C and D) Divergence of viral RNA sequence. Phylogenic trees (C) and genetic diversity (D) of *env* ORF sequences in the plasma of infected mice are shown. In panel C, the scale bar indicates the number of substitutions per site. The amplicons harboring statistically significant levels of G-to-A mutations (*P*<0.05 by Fisher's exact test using Hypermut 2.0) are indicated by asterisks.

Our findings in both *in vivo* (Figure 4F) and *in vitro* (Figure 4G) demonstrated that APOBEC3G prefers to target T*G*GG as substrate. Importantly, T*G*G and T*A*G are the codons encoding Tryptophan and termination codon, respectively, suggesting that APOBEC3G can readily cause lethal mutations (i.e., T*G*G-to-T*A*G termination mutations). On the other hand, APOBEC3F and APOBEC3D generated *G*AA-to-*A*AA and *G*A-to-*A*A mutations, respectively (Figures 4F and 4G), which do not generate termination codons and thus cause lethal mutations less frequently. These findings raised a hypothesis that APOBEC3G directly causes lethal mutations, while APOBEC3F and APOBEC3D induce the accumulation of nonsynonymous mutations in the viral genome. To address this hypothesis, single genome sequencing (SGS) assays [50] were performed using viral RNA isolated from the plasma of infected mice at 6 wpi. Since G-to-A mutations were frequently observed in the proximal upstream region of the 3′ polypurine tract (positioned at 9056–9071; Figure S6), which was consistent with previous reports [49], [51], we focused on the *env* open reading frame (ORF) sequence. As shown in Figure 5A (the raw data is shown in Figure S7), SGS assay revealed that G-to-A mutations were frequently observed in the viral RNA genomes of 4A HIV-1-infected mice but not of WT, 5A, and 4A5A HIV-1-infected mice. In addition, in the 91 *env* amplicons of 4A HIV-1-infected mice, 37 analyzed amplicons harbored more than 10 G-to-A mutations, and 15 analyzed amplicons harbored more than 10 *G*A-to-*A*A mutations, respectively (Figure S8). On the other hand, the amplicons harboring G-to-A hypermutations were rarely detected in WT, 5A, and 4A5A HIV-1-infected mice (Figure S8). Moreover, although termination mutations were prominently detected in the proviral DNA of 5A and 4A5A HIV-1-infected mice (Figures 4D and 4E), the percentages of termination mutation in the viral RNA in plasma of 5A and 4A5A HIV-1-infected mice were comparable to that of 4A HIV-1-infected mice (Figure 5B; 4A HIV-1 versus 5A HIV-1, *P* = 0.06; 4A HIV-1 versus 4A5A HIV-1, *P* = 0.19 by Chi-square test for independence). These findings strongly suggest that APOBEC3G-mediated G-to-A mutations frequently result in lethal mutations.

Interestingly, the phylogenic trees displayed that the *env* sequences in 4A HIV-1-infected mice were highly divergent and harbored significant levels of G-to-A mutations (Figure 5C). Furthermore, the analyses on genetic distance directly demonstrated that the *env* RNA sequences of 4A HIV-1-infected mice were highly divergent when compared to those of WT, 5A, and 4A5A HIV-1-infected mice (Figure 5D). Taken together, these findings provide strong evidence that APOBEC3F and APOBEC3D have the potential to restrict HIV-1 propagation, but at the same time, can also augment the emergence of quasispecies through sub-lethal G-to-A mutations *in vivo*.

### Emergence of CCR5/CXCR4 dual-tropic HIV-1 in 4A HIV-1-infected mice

**Figure 6:**
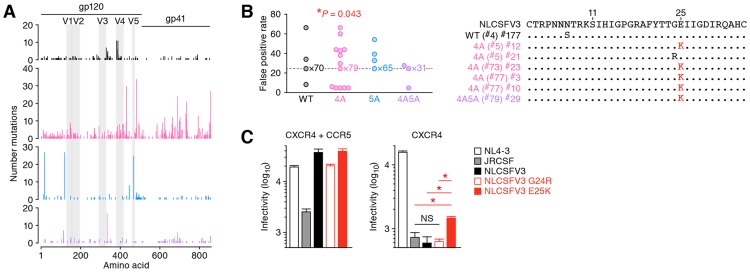
Functional evolution of 4A HIV-1 *in vivo*. (A) Mutations in *env* ORFs. (B) Estimation of coreceptor usage. Putative coreceptor usage was determined by using a geno2pheno coreceptor algorithm [54] as described in Materials and Methods. Mouse IDs are shown in parentheses (correspond to those in Table S2). The frequency of putative CXCR4-tropic HIV-1 in 4A HIV-1-infected mice was significantly higher than those in the mice infected with the other viruses (*P* = 0.043 by Chi-square test for independence). (C) Functional evaluation of coreceptor usage. Viral infectivity was measured by MaRBLE assay using R5-MaRBLE cells (*left*) and X4-MaRBLE cells (*right*). The data represents average with SD. The assay was performed in triplicate. Asterisks represent statistically significant differences (*P*<0.05 by Student's *t* test). NS, no statistical significance.

As shown in Figure 6A, mutations were detected in both conserved and variable regions of *env*. Previous studies have demonstrated that the variable region 3 (V3) of *env*, particularly the residues positioned at 11 and 25 in the V3, determines the CCR5 or CXCR4 coreceptor usage for HIV-1 entry [52], [53]. Since we detected the diversified *env* sequences particularly in 4A HIV-1-infected mice (Figure 5C), we hypothesized the emergence of viruses that can use CXCR4 as the coreceptor in 4A HIV-1-infected mice. To address this possibility, we screened putative CXCR4-tropic HIV-1 by using a geno2pheno tool, which predicts the coreceptor usage based on nucleotide sequence [54], and found that the frequency of putative CXCR4-tropic HIV-1 in 4A HIV-1-infected mice was significantly higher than those in mice infected with WT, 5A, and 4A5A HIV-1s (Figure 6B, *left*). The detected putative CXCR4-tropic viruses were a N7S mutant from a WT HIV-1-infected mouse, a G24R mutant from a 4A HIV-1-infected mouse, and five E25K mutants from three 4A HIV-1-infected and one 4A5A HIV-1-infected mice (Figure 6B, *right*). It was particularly noteworthy that the E25K mutant detected was due to *G*AA-to-*A*AA mutation, which is the mutation signature mediated by APOBEC3F and APOBEC3D. To functionally evaluate whether these mutants can use CXCR4 as the coreceptor, we prepared the mutated virus based on NLCSFV3, which exclusively use CCR5 as the coreceptor. As shown in Figure 6C, we directly demonstrated that the infectivity of E25K mutant in CXCR4^+^ MaRBLE cells was 2.5-fold higher than that of parental NLCSFV3 with a statistical significance (*P* = 0.0073). Taken together, these findings strongly suggest that the G-to-A mutation mediated by APOBEC3F and APOBEC3D can contribute to the conversion of viral coreceptor usage from CCR5 to CXCR4.

## Discussion

Previous studies including ours have demonstrated that endogenous APOBEC3 proteins have robust potential to diminish HIV-1 replication in humanized mouse models [23], [41]. Furthermore, Krisko et al. have demonstrated that greater than 80% of G-to-A mutations in their *in vivo* experiments were in the context of *G*G-to-*A*G mutations, suggesting that endogenous APOBEC3G is the dominant restricting factor *in vivo* [41]. However, there are no reports that directly evaluate and compare the sole effects of endogenous APOBEC3G and/or APOBEC3D/F on HIV-1 replication *in vivo*. In addition, these papers [23], [41] did not explore the possibility that endogenous APOBEC3 protein(s) may contribute to viral diversification. In our present study, we directly examined these two issues by using 3 kinds of HIV-1 *vif* mutants and a humanized mouse model. We demonstrated that endogenous APOBEC3G and APOBEC3D/F are intrinsic restriction factors against HIV-1. Moreover, we observed that endogenous APOBEC3D and APOBEC3F are capable of enhancing viral diversification *in vivo*.

We found that the propagation of HIV-1 *vif* mutants, particularly 5A and 4A5A, was severely suppressed even at high doses (Figures 2A–2D and S2). Consistent with previous reports [42], [43], endogenous *APOBEC3G* was highly expressed in the splenic human CD4^+^ T cells of humanized mice when compared to *APOBEC3F* and *APOBEC3D* (Figure 3B). In addition, the proviral DNA of 5A and 4A5A HIV-1-infected mice exhibited T*G*GG-to-T*A*GG hypermutations (Figure 4F), and APOBEC3G preferentially targeted to the tetranucleotide T*G*GG as substrate (Figure 4G), which readily results in termination codon mutations (Figures 4D and 4E). These findings indicate that endogenous APOBEC3G is an intrinsic factor that severely restricts HIV-1 propagation *in vivo*.

It was notable that the proviral DNA (Figures 4B–4F) and the *vif* ORF in the spleens of 4A5A HIV-1-infected mice (Figure S3) exhibited the signature of APOBEC3G-mediated mutations. In this regard, an *in silico* study has been recently reported that APOBEC3G and APOBEC3F rarely co-mutate the same viral genome in infected individuals [55]. Because the expression level of *APOBEC3G* was higher than those of *APOBEC3F* and *APOBEC3D* (Figure 3B), our findings suggest that APOBEC3G more predominantly affects HIV-1 replication *in vivo* than APOBEC3F and APOBEC3D.

When compared to APOBEC3G, the potential role of APOBEC3D and APOBEC3F in inhibition of viral replication has been controversial. Refsland and colleagues have recently demonstrated the anti-HIV-1 ability of APOBEC3D and APOBEC3F endogenously expressed in a human CD4^+^ T cell line called CEM2n cells [15]. On the other hand, certain previous studies using human PBMC *in vitro* cultures have suggested that endogenously expressed APOBEC3F moderately restricts [56] or does not restrict [16] *vif*-deficient HIV-1 replication. In this regard, it should be noted that *in vitro* culture conditions use human CD4^+^ T cell lines and/or human PBMCs artificially activated with mitogens such as phytohemagglutinin, which may not exactly mimic *in vivo* conditions, and therefore, may not reproduce the expression levels of APOBEC3D and APOBEC3F *in vivo*. Thus, it was important to carry out the *in vivo* experiments, which now firmly establish that APOBEC3D/F do indeed exert a substantial anti-viral effect on HIV-1 replication (Figures 2A–2D).

Although we demonstrated that the growth kinetics of 4A HIV-1 was significantly impaired compared to WT HIV-1 (Figures 2A–2D), the kinetics of 4A HIV-1 varied in each mouse (Figure 3A) and were significantly higher than those of 5A and 4A5A HIV-1 (Figures 2C and 2D). Also, the growth kinetics of 4A HIV-1 significantly and negatively correlated to the expression level of *APOBEC3F* but not *APOBEC3D* (Figure 3D), suggesting that endogenous APOBEC3F more critically modulates 4A HIV-1 replication *in vivo* than APOBEC3D. In fact, endogenous expression level of *APOBEC3F* was higher than that of *APOBEC3D* (Figure 3B). Moreover, anti-HIV-1 activity of APOBEC3F was higher than that of APOBEC3D in *in vitro* transfection experiments (Figures 1C and S1), which are consistent with previous reports [13], [17], [48]. Therefore, these results suggest that the growth kinetics of 4A HIV-1 is predominantly impaired by APOBEC3F rather than APOBEC3D.

It is known that certain APOBEC3 proteins can impair HIV-1 replication by inhibiting viral reverse transcription (RT) independently of their deaminase activities [57]–[60]. In this regard, based on an experimental-mathematical approach, we have recently demonstrated that APOBEC3G restricts HIV-1 replication almost completely in a deaminase activity-dependent manner, while APOBEC3F impairs viral replication with the combination of G-to-A mutations and inhibition of viral RT [61]. In addition, although a deaminase-defective APOBEC3G mutant (E259Q) severely lost its anti-viral effect by 173-fold, the anti-viral effect of the deaminase-defective APOBEC3D (E264Q) and APOBEC3F (E251Q) differed only 2–3-fold compared to the WT proteins (Figure S1). These findings suggest that the anti-viral effect of APOBEC3D and APOBEC3F may be partially attributed to their deaminase-independent properties. On the other hand, Albin et al. have recently reported that *vif*-deficient HIV-1 can overcome the anti-viral effect of deaminase-defective APOBEC3F in a spreading infection experiment using T cell lines, and that APOBEC3F's deaminase activity is crucial for long-term restriction of *vif*-deficient HIV-1 replication [62]. Moreover, Mbisa et al. previously reported that virion-incorporated APOBEC3F and APOBEC3G potently inhibit HIV-1 integration [63]. Thus, these findings indicate that APOBEC3 proteins potently suppress HIV-1 replication by at least 3 different modes: (i) G-to-A mutation; (ii) inhibition of viral RT; and (iii) inhibition of viral integration; moreover, the magnitude of each mode of inhibition may be different for specific APOBEC3 proteins. Because APOBEC3's anti-viral modes are complex and intertwined, it would be technically impossible to quantitatively elucidate this under *in vivo* conditions. However, when compared to the mutation signature of APOBEC3G (T*G*GG-to-T*A*GG), APOBEC3D and APOBEC3F respectively preferred the dinucleotide (*G*A) and trinucleotide (*G*AA), which rarely led to stop codon mutations (Figure 4G) [61]. Although the extent of deaminase-dependent anti-HIV-1 activity of APOBEC3 proteins *in vivo* remains undetermined, our results suggest that endogenous APOBEC3D and APOBEC3F may inhibit HIV-1 replication *in vivo* in a manner that is less dependent on their deaminase activity than APOBEC3G.

Separate from the anti-HIV-1 ability of APOBEC3 proteins, some papers have suggested that the mutations generated by APOBEC3 proteins, particularly APOBEC3G, can promote viral evolution [18], [19], [21]. In this regard, it was particularly noteworthy that the viral RNA sequences in the plasma of 4A HIV-1-infected mice were highly diversified when compared to those of WT, 5A, and 4A5A HIV-1-infected mice (Figures 5C and 5D). These findings suggest that the G-to-A mutations mediated by APOBEC3D and APOBEC3F, but not by APOBEC3G, can increase the genetic diversity of viral populations. In fact, here we directly showed the emergence of CCR5/CXCR4 dual-tropic HIV-1 most exclusively in 4A HIV-1-infected mice (4 out of the 91 amplicons analyzed; Figure 6C, *right*), and the 4 E25K amplicons detected had intact ORFs (i.e., no termination mutations in the amplicon). More importantly, the E25K mutant in the V3 region of *env*, a coreceptor-switched HIV-1, was generated by a *G*AA-to-*A*AA mutation, strongly suggesting that this mutation may be caused by APOBEC3F, and also possibly APOBEC3D. Regarding viral coreceptor usage, it is well known that the charge of two specific amino acids in the V3 region of HIV-1 Env, positioned at 11 and 25, strongly influence the coreceptor usage [52], [53]. Our findings strongly suggest that one of the two crucial mutations, E25K, needed for conversion of CCR5 to CXCR4 usage, is facilitated by APOBEC3D/F. This makes it more likely that coreceptor conversion will occur as a result of a random RT error leading to a substitution at the position 11 in genomes that have the APOBEC3D/F-associated mutation.

In addition to the conversion of coreceptor usage (Figure 6C), we found that the sites preferred by APOBEC3D and APOBEC3F may potentially lead to the resistance to anti-HIV-1 drugs (Table S1). Furthermore, our results suggest that 4A HIV-1 can propagate *in vivo* when APOBEC3F expression level was relatively low (Figure 3D). Our data further suggest that sub-lethal G-to-A mutations caused by endogenous APOBEC3D and APOBEC3F, which are expressed at a lower level, rather than APOBEC3G, can lead to diversification of HIV-1 genomes leading to increased viral variation and evolutionary potential. Although our viruses used in this study produce defective Vifs, we believe it reflects the natural infection because Simon et al. have shown that defective *vif*s are often seen during natural infection in patients [64]. Thus, the types of mutations and diversification we observed in this study would be quantitatively higher, but similar to the diversification that occurs during natural infection, as a result of the emergence of *vif*-mutated viruses.

In conclusion, here we demonstrated that endogenous APOBEC3G is the *bona fide* anti-HIV-1 restriction factor even *in vivo*. On the other hand, we also provide strong evidence indicating that endogenous APOBEC3D and APOBEC3F suppress viral replication *in vivo*, while these proteins potently induce viral evolution. These findings suggest that the impairment of Vif-APOBEC3G interaction can be a novel target for anti-HIV-1 drugs, while the restoration of deaminase activity of APOBEC3D and APOBEC3F by inhibiting Vif-mediated degradation may potentially lead to the enhancement of viral diversification. As shown in Figure 1B, both DRMR and YRHHY motifs are exposed on the surface of Vif protein [39]. Therefore, it may be possible to design compounds that target the YRHHY motif and specifically block Vif-APOBEC3G interaction, which may be ideal candidates for development of novel anti-HIV-1 drugs.

## Materials and Methods

### Ethics statement

All procedures including animal studies were conducted following the guidelines for the Care and Use of Laboratory Animals of the Ministry of Education, Culture, Sports, Science and Technology, Japan. The authors received approval from the Institutional Animal Care and Use Committees (IACUC)/ethics committee of Kyoto University institutional review board (protocol number D13-25). All protocols involving human subjects were reviewed and approved by the Kyoto University institutional review board. Informed written consent from human subjects was obtained in this study.

### Humanized mice

NOG mice [65] were obtained from the Central Institute for Experimental Animals (Kawasaki, Kanagawa, Japan). The mice were maintained under specific-pathogen-free conditions and were handled in accordance with the regulation of IACUC/ethics committee of Kyoto University. Human CD34^+^ hematopoietic stem cells were isolated from human fetal liver as previously described [66]. The humanized mouse (NOG-hCD34 mouse) was constructed as previously described [22]–[27]. Briefly, 82 newborn (aged 0 to 2 days) NOG mice from 19 litters were irradiated with X-ray (10 cGy per mouse) by an RX-650 X-ray cabinet system (Faxitron X-ray Corporation) and were then intrahepatically injected with the obtained human fetal liver-derived CD34^+^ cells (8×10^4^ to 17×10^4^ cells). A list of the humanized mice used in this study is summarized in Table S2.

### Cell culture

293T cells and TZM-bl cells (obtained through the NIH AIDS Research and Reference Reagent program) [67] were maintained in DMEM containing 10% fetal calf serum (FCS) and antibiotics. X4-MaRBLE and R5-MaRBLE cells (kindly provided by Dr. Wataru Sugiura) [68] were maintained in RPMI 1640 containing 10% FCS and antibiotics. For X4-MaRBLE cells, 250 µg/ml Geneticin and 0.1 µg/ml Puromycin were added to the culture medium. For R5-MaRBLE cells, 150 µg/ml Hygromycin B, 250 µg/ml Geneticin, and 0.1 µg/ml Puromycin were added to the culture medium.

### Virus preparation and infection

IMCs of CCR5-tropic HIV-1 (strain NLCSFV3) [40] and its derivatives were constructed based on pNLCSFV3 [40]. To construct pNLCSFV3-DRMR/AAAA (pNLCSFV3-4A), pNLCSFV3-YRHHY/AAAAA (pNLCSFV3-5A), and pNLCSFV3*Δvif*, the *Age*I-*EcoR*I fragments of pNL4-3-based these mutants [17], [38], [69] and pNL4-3*Δvif* [70] were subcloned into the *Age*I-*EcoR*I site of pNLCSFV3 [40]. To construct pNLCSFV3-4A5A, the 5A mutation was inserted into pNLCSFV3-4A as previously described [69]. The sequences of these constructed plasmids were confirmed by sequencing PCR. To prepare the virus solutions for the experiments using humanized mice, 30 µg of pNLCSFV3 or its derivatives (pNLCSFV3-4A, pNLCSFV3-5A, or pNLCSFV3-4A5A) was transfected into 293T cells by the calcium-phosphate method as previously described [23], [25]. After 48 h posttransfection, the culture supernatant was harvested, centrifuged, and then filtrated through a 0.45-µm filter (Millipore) to produce virus solution. The amount of virus particles was quantified by using an HIV-1 p24 antigen ELISA kit (Zeptometrix), and 50% infectious dose (ID_50_) was measured by Reed-Meunch's method as previously described [25]. Virus solutions containing 5 ng (Figure 2), 50 ng and 500 ng (Figure S2) of p24 antigen (equivalent to 1,500, 15,000, and 150,000 ID_50_, respectively) were intraperitoneally inoculated into NOG-hCD34 mice. RPMI 1640 was used for mock infection.

### Peripheral blood collection, mononuclear cell isolation, and quantification of HIV-1 RNA in plasma

Peripheral blood and plasma were collected at 0, 1, 2, 3, 5, and 6 wpi as previously described [22], [23], [25], [26]. The mice were sacrificed at 6 wpi with anesthesia, and the spleen was crushed, rubbed, and suspended as previously described [22], [23], [25], [26]. To obtain splenic human MNCs, the splenic cell suspension was separated by using Ficoll-Paque (Pharmacia) as previously described [22], [23], [25], [26]. The amount of HIV-1 RNA in 50 µl plasma was quantified by Bio Medical Laboratories, Inc. (the detection limit of HIV-1 RNA is 800 copies/ml).

### Flow cytometry and hematometry

Flow cytometry was performed with a FACS Canto II (BD biosciences) as previously described [22], [23], [25], [26], and the obtained data were analyzed with Cell Quest software (BD biosciences) and FlowJo software (Tree Star, Inc.). For flow cytometry analysis, anti-CD45-PE (HI30; Biolegend), anti-CD3-APC-Cy7 (HIT3a; Biolegend), and anti-CD4-APC (RPA-T4; Biolegend) antibodies were used. Hematometry was performed with a Celltac α MEK-6450 (Nihon kohden, Co.) as previously described [23], [25], [26].

### Transfection, western blotting, TZM-bl assay, and MaRBLE assay

*In vitro* transfection experiments were performed by using Lipofectamine 2000 (Life technologies) according to the manufacture's protocol. After 48 h posttransfection, the culture supernatant was harvested, centrifuged, and then filtrated through a 0.45-µm filter (Millipore) to produce virus solution. For the experiments shown in Figure 1C, 2 µg of pNLCSFV3 or its derivatives (pNLCSFV3-4A, pNLCSFV3-5A, or pNLCSFV3-4A5A) was cotransfected with 100 ng of flag-tagged APOBEC3D, APOBEC3F, or APOBEC3G expression plasmid into 293T cells. For the experiments shown in Figures 2E and 2F, 500 ng of pNLCSFV3*Δvif* and 500 ng of Vif expression plasmids (see below) were cotransfected with 50 ng of flag-tagged APOBEC3F or APOBEC3G expression plasmid [17] into 293T cells. For the experiments shown in Figure 4G, 2 µg of pNLCSFV3*Δvif* was cotransfected with 100 µg of flag-tagged APOBEC3D, APOBEC3F, or APOBEC3G expression plasmid into 293T cells. The virus solutions were prepared as described above. Then, the virus solutions were treated with DNase I (50 unit; Takara) at 37°C for 1 h and inoculated into TZM-bl cells. The infected TZM-bl cells were harvested at 18 h postinfection and DNA was extracted as described below. Western blotting was performed as previously described [23], [25], and anti-Vif antibody (clone #2221; obtained through the NIH AIDS Research and Reference Reagent program) and anti-α-Tubulin (TUBA) monoclonal antibody (DM1A; Sigma) were used. To quantify the infectivity of virus solution, TZM-bl assay was performed as previously described [23], [25]. MaRBLE assay was performed as previously described [68] with minor modifications. Briefly, the virus solutions (normalized to the amount of p24 antigen) were inoculated into X4-MaRBLE or R5-MaRBLE cells (1×10^5^ cells). At 72 h postinfection, the cells were harvested, and the luciferase activity was measured as previously described [71].

### PCR, RT-PCR, and real-time RT-PCR

DNA and RNA were extracted from the splenic human MNCs at 6 wpi or infected TZM-bl cells as previously described [23], [25]. cDNA was prepared by using SuperScript III reverse transcriptase (Life technologies) with DNase I (Life technologies), RNaseOUT (Life technologies), and random primers according to the manufacture's procedure. To amplify *vif* ORF (Figures 2E, 2F, and S3), RT-PCR was performed by using PrimeSTAR GXL DNA polymerase (Takara) according to the manufacture's protocol, and the following primers were used: Vif-fwd (4929–4948), 5′-gtt tgg aaa gga cca gca aa-3′; and Vif-rev (5703–5722), 5′-gcc caa gta tcc ccg taa gt-3′. To analyze the sequence of full-length proviral DNA (Figures 4B–4E and S6), PCR was performed by using *Pfu* Ultra II DNA polymerase (Stratagene) according to the manufacture's protocol, and the following primers were used: 5′ region (475–1698, 1,224 bp), 5LTRF#1 (455–474), 5′-ggt ctc tct ggt tag acc ag-3′; and 5LTRR#1 (1699–1718), 5′-gaa gct tgc tcg gct ctt ag-3′; 5′/central region (1342–3530, 2,189 bp), 5F#7 (1322–1341), 5′-gag cca ccc cac aag att ta-3′; and 5/cR#7 (3531–3550), 5′-tgc ccc tgc ttc tgt att tc-3′; central/3′ region (3420–5888, 2,469 bp), C#3F (3400–3419), 5′-ggg gaa cca aag cac taa ca-3′; and 5R#1 (5889–5913), 5′-ttt aca ata gca att ggt aca agc a-3′; 3′ region (5453–9526, 4,074 bp), 3F#2 (5428–5452), 5′-agt cct agg tgt gaa tat caa gca g-3′; and 3LTRR#1 (9527–9547), 5′-ctg gtc taa cca gag aga cc-3′. The products of PCR and RT-PCR were cloned into pCRII-blunt-TOPO by using Zero blunt TOPO PCR cloning kit (Life technologies) according to the manufacture's protocol. To prepare the expression plasmids of the Vif mutants (Figures 2E and 2F), the pCRII-blunt-TOPO containing *vif* ORFs were digested with *EcoR*I and blunted. The obtained DNA fragments containing *vif* ORF were subcloned into the *Hpa*I site of pDON-AI (Takara). Real-time RT-PCR was performed as previously described [23]. Briefly, *APOBEC3D, APOBEC3F, APOBEC3G* [43] and *IFNB* [72] were amplified by using the primers previously reported. The primers for *GAPDH* were purchased from Life technologies. The expression levels of *APOBEC3D, APOBEC3F*, and *APOBEC3G* (Figure 3C) were standardized as previously described [42], [43]. To construct pNLCSFV3 G24R and E25K (Figure 6C), the DNA sequences containing *env* G24R or E25K mutations were digested with *Mlu*I and *Xba*I, and the resultant DNA fragments were subcloned into the *Mlu*I-*Xba*I site of pNLCSFV3.

### SGS assay

SGS assay was performed as previously described [50]. Briefly, viral RNA was extracted from the plasma (100 µl) of infected mice at 6 wpi by using QIAamp viral RNA mini kit (Qiagen), and cDNA was prepared as previously described [50].

### Sequencing PCR

Sequencing PCR was performed as previously described [23], and the sequence data were analyzed by Seqscape software v2.5 (Applied Biosystems) and Sequencher software (Hitachi). To analyze the sequence of *vif* (Figures 2E, 2F and S3), M13 primers were used. To analyze the sequence of full-length proviral DNA (Figures 4B–4G and S6), M13 primers and the following primers were used: 5#6 (1609–1633), 5′-gta aga atg tat agc cct acc agc a-3′; Cl#1 (2178–2197), 5′-cag gtt tgg gga aga gac aa-3′; C#2 (2700–2719), 5′-ggg cct gaa aat cca tac aa-3′; C#4 (4004–4023), 5′-ttt gca gga ttc ggg att ag-3′; C#5 (4499–4518), 5′-agc aga gac agg gca aga aa-3′; C#6 (5058–5077), 5′-ggt gat gat tgt gtg gca ag-3′; 3#1 (5960–5979), 5′-gca tct cct atg gca gga ag-3′; 3#2 (6651–6660), 5′-gcg gga gaa tga taa tgg ag-3′; 3#3 (7315–7334), 5′-ccc aga aat tgt aac gca ca-3′; 3#4 (7947–7966), 5′-gaa tcc tgg ctg tgg aaa ga-3′; 3#5 (8511–8530), 5′-gct acc acc gct tga gag ac-3′; and 3#6 (8969–8988), 5′-gga gga aga ggt ggg ttt tc-3′. For SGS assay (Figure 5), direct sequencing was performed by using the primers used in the 2nd SGS PCR and the primers 3#2 and 3#3.

### Semiquantitative 3D-PCR

Semiquantitative 3D-PCR (Figure 4A) was performed as previously described [48]. Briefly, we used the following primers according to the previous report [48]: 1st-fwd (2723–2746), 5′-tcc art att trc cat aaa raa aaa-3′; 1st-rev (3575–3598), 5′-tty aga ttt tta aat ggy tyt tga-3′; 2nd-fwd (3023–3049), 5′-aat att cca rtr tar cat rac aaa aat-3′; and 2nd-rev (3561–3586), 5′-aat ggy tyt tga taa att tga tat gt-3′. The 1st PCR products were quantified, and constant amounts were used for secondary PCR over 87.5 to 77.0°C range of denaturation temperatures. The 2nd PCR products were run on agarose gels and were visualized by staining with ethidium bromide.

### Sequence data analysis and bioinformatics

To analyze the diversity of *vif* in HIV-1 group M (Figure 1A), we obtained 7,118 *vif* ORF sequences registered in Los Alamos HIV sequence database (http://www.hiv.lanl.gov). The 7,118 datasets were aligned by using ClustalW [73] implemented in MEGA 5.1 software [74], and the logo plot shown in Figure 1A was generated by using WebLogo 3 (http://weblogo.threeplusone.com/). To analyze the effect of G-to-A mutation in proviral DNA (Figures 4D and 4E), the sequences of all viral genes (*gag, pol, vif, vpr, tat, rev, vpu, env, and nef*) were obtained from the sequence of full-length proviral DNA (Figure 4B), and the codon-based alignments were constructed using a Gene Cutter tool from the Los Alamos HIV sequence database (http://www.hiv.lanl.gov/content/sequence/GENE_CUTTER/cutter.html). The effect of G-to-A mutation in *env* ORF of viral RNA (Figure 5B) was also analyzed as descried above. To analyze APOBEC3-mediated mutations (Figures 4F and 4G), hypermut 2.0 (http://www.hiv.lanl.gov/content/sequence/HYPERMUT/hypermut.html) was used. The *env* ORF sequences obtained by SGS (see above) were aligned by using ClustalW [73] implemented in MEGA 5.1 software [74]. The sequence of WT NLCSFV3 *env* was used as outgroup. The best fitting substitution model was determined using jModelTest 2.1.3. [75]. The Akaike information criterion (AIC) implemented in jmodeltest-2.1.3 selected GTR+I+G as the best-fit. Since this model is not available in MEGA 5.1 software, the TrN+I+G [76], the second best-fit model, was used in further analyses. Genetic distances among *env* ORF sequences (Figure 5D) were calculated with MEGA 5.1 software under the Tamura-Nei model [76]. ML phylogenetic trees (Figure 5C) were reconstructed using PhyML-3.1 under TN93 model [76] with 1,000 bootstrap resamplings [77]. The *env* V3 sequences determined by SGS were used for the genotypic coreceptor usage prediction based on an algorithm, geno2pheno coreceptor [54]. The original g2p coreceptor model was selected, and the sequences below the 10% false-positive rate cutoff were defined as putative CXCR4-tropic viruses (Figure 6B). The major drug resistance sites, which are potentially induced by APOBEC3D and APOBEC3F (Table S1), were determined based on the current IAS-USA lists [78]. The sequence of HIV-1 strain HXB2 (Genbank accession number: FB707281) were used as reference.

### 3D structure of Vif

The 3D structure of Vif (Figure 1B) was generated on PyMOL v1.6 (http://www.pymol.org/) with the crystal structure of Vif-CBFβ-CUL5-ELOB-ELOC complex (PDB code: 4N9F) [79].

### Calculation of AUC of VL

The AUC (Figure 2C) was calculated from the VL data using the trapezoidal rule. For example, let us define that *V(t)* is a VL at time *t*. Then the AUC from 0 to 6 wpi is calculated as follows:
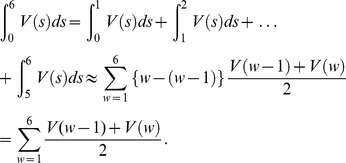



### Estimation of virus replication rate

To quantify the dynamics during acute virus infection (Figure 2D), we used a recently developed model describing the loss of target cells phenomenologically as follows:
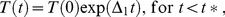
(1)

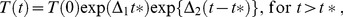
(2)

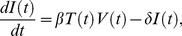
(3)

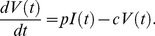
(4)Modeling HIV infection it is well accepted to make the quasi-steady assumption, *dV*(*t*)/*dt* = 0, and to write that *V*(*t*) = p*I(*t*), where p* is a scaled production parameter (p* = *p/c*). Because we are fitting VLs, *V*(*t*), rather than number of infected cells, *I*(*t*), we substitute *I*(*t*) = *V*(*t*)/p* into Eq. (3) to obtain
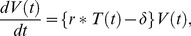
(5)where r* = p*β is the viral replication rate per target cell, and *δ* remains the death rate of infected cells. Because the number of target cells seems to decrease exponentially in phases during the acute phase of several virus infections, we approximated the dynamics of target cells by a piece-wise exponential function. The parameters Δ_1_ and Δ_2_ represent the two daily loss rates of target cells, and t* represents the time at which the function switches slope. Because Eqs. (1), (2), and (5) define a non-autonomous linear differential equation, we derived the following analytical solution describing the acute phase of virus infections:
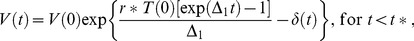
(6)

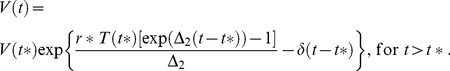
(7)We employed the solution of Eqs. (1), (2), (6), and (7) to fit the 6-week time courses of VLs and target cells as shown in Figures 2 and S2 (using the FindMinimum package of *Mathematica 9.0* to minimize the sum of squared residuals). This model has 6 parameters: *T*(0), Δ_1_, Δ_2_, *V*(0), r*, and *δ*. The first 3 parameters, *T*(0), Δ_1_, and Δ_2_, are estimated from the observed number of peripheral CD4^+^ T cells per ml of blood. For the latter 3 parameters, *V*(0), r*, and *δ*, we fix *δ* = 1 per day [80], [81], because this is general estimate for the death rate of productively infected cells. The initial value of VL, *V*(0), was set to the detection limit of the assay (800 copies/ml plasma). The replication rate, r*, was estimated from the data.

### Statistical analysis

Data were presented as averages ± SEMs or SDs. Statistical differences were determined by Student's *t* test (Figures 1C, 2A–2D, 3C, and 6C), Chi-square test for independence (Figures 4D–4G, 5B, 6B, and S5), and Fisher's exact test (Figure 5C). To determine statistically significant correlation (Figures 3D and S4), Pearson correlation coefficient (*r*) was applied.

### Accession numbers

The GenBank (http://www.ncbi.nlm.nih.gov/genbank/) accession numbers for the genes mentioned in the text are as follows: *APOBEC3D* (NM_152426), *APOBEC3F* (NM_145298), *APOBEC3G* (NM_021822), *IFNB* (NM_002176), and *GAPDH* (NM_002046).

### Online supplemental material

Figure S1 shows the anti-viral activity of WT and catalytically inactive APOBEC3 proteins *in vitro*. Figure S2 shows the dynamics of WT HIV-1 and HIV-1 *vif* mutants infection in humanized mice at higher doses. Figure S3 shows the summary of mutations in *vif* ORF. Figure S4 shows the correlation of *APOBEC3* and *IFNB* expressions. Figure S5 shows the statistical analyses on the preferential G-to-A mutation sites. Figure S6 shows the summary of mutations in the proviral DNA of infected humanized mice. Figure S7 shows the raw data of SGS assay. Figure S8 shows the extent of mutation in each amplicon of viral *env*. Table S1 shows putative drug-resistance mutations potentially induced by APOBEC3D and APOBEC3F. Table S2 shows the list of humanized mice used in this study.

## Supporting Information

Figure S1 **Anti-viral activity of WT and mutated APOBEC3 proteins** ***in vitro*** **.** Two micrograms of pNLCSFV3*Δvif* was cotransfected with 100 ng of flag-tagged APOBEC3 expression plasmid into 293T cells. (A) Western blotting. The input of cell lysate was standardized to α-Tubulin (TUBA), and representative results are shown. (B) TZM-bl assay. The infectivity of released virus was determined by using TZM-bl cells. The infectivity of each virus is normalized to the value of no APOBEC3. The assay was performed in triplicate. The data represents average with SD.
(TIF)

Figure S2 **Dynamics of WT HIV-1 and HIV-1** ***vif*** **mutants infection in humanized mice at higher doses.** (A and B) Virus solutions containing 50 ng (A; 4A HIV-1 [n = 8], 5A HIV-1 [n = 8], and 4A5A HIV-1 [n = 6]) or 500 ng (B; 4A HIV-1 [n = 5], 5A HIV-1 [n = 8], and 4A5A HIV-1 [n = 6]) p24 antigens were intraperitoneally inoculated into humanized mice. The amount of viral RNA in plasma (*top*) and the level of peripheral CD4^+^ T cells (CD45^+^ CD3^+^ CD4^+^ cells) (*bottom*) were analyzed at 0, 1, 2, 3, 5, and 6 wpi. The averages are shown in circles with SEMs, and the values from each mouse are shown by line. In panel A, the detection limit of HIV-1 RNA is 800 copies/ml plasma.
(TIF)

Figure S3 **Mutations in** ***vif*** **ORF.** The *vif* ORFs (5041–5619, 579 bases) of viral RNA in the spleen of infected mice (WT, n = 82 from 3 mice; 4A, n = 320 from 7 mice; 5A, n = 132 from 3 mice; and 4A5A, n = 59 from 1 mouse) were sequenced. (A) The mutation matrix (*left*) and the pie chart of G-to-A mutation (*right*) are shown. In the *right* panel, the diameters of pie charts represent the percentage of G-to-A mutations in total mutations. (B) The extent of mutation in each amplicon of *vif* ORF sequences. The numbers of total mutations (*top*, gray), G-to-A mutations (*upper middle*, black), GA-to-AA mutations (*lower middle*, red), and GG-to-AG mutations (*bottom*, blue) within each amplicon are respectively shown. (C) The *vif* amplicons harboring nonsynonymous mutations are summarized. “X” means stop codon mutation. The *vif* ORFs indicated by asterisks were used for the functional assay, and the results are shown in Figures 2E and 2F. In the panel of 4A5A, the amplicon harboring both R132K and I124M mutations (indicated by double daggers) were frequently detected and were used for the functional assay (Figures 2E and 2F).
(TIF)

Figure S4 **Correlation of** ***APOBEC3*** **and** ***IFNB*** **expressions.** The mRNA expression levels of *APOBEC3D*, *APOBEEC3F*, *APOBEC3G*, and *IFNB* in the splenic human CD4^+^ T cells of humanize mice (n = 73) were measured by real-time RT-PCR. The expression level of each gene was normalized to that of *GAPDH* and was shown as relative expression. The correlation between each *APOBEC3* (A) and between *APOBEC3* (x-axes) and *IFNB* (y-axes) (B) are respectively shown. The lines represent exponential approximation. Pearson correlation coefficient (*r*) was adopted to determine statistically significant correlation between each value.
(TIF)

Figure S5 **Statistical analyses on the preferential G-to-A mutation sites.** The detected G-to-A mutation sites in the proviral DNA of *vif*-mutated HIV-1-infected mice (A) and *in vitro* experiments (B) were classified according to the nucleotides positioned between −5 to +5 from the detected G-to-A mutation sites (position 0; see also Figures 4F and 4G). Statistical differences at each position were determined by Chi-square test for independence, and the *P* values at each position is shown in y-axes. Statistically significant differences (*P*<0.001) are shown with asterisks.
(TIF)

Figure S6 **Mutations in the proviral DNA of infected humanized mice.** The percentages of mutations in each site are summarized.
(TIF)

Figure S7 **Raw data of SGS assay.** The *env* ORF (6221–8782, 2,562 bases) of viral RNA in the plasma of infected mice (WT, n = 73 from 2 mice; 4A, n = 91 from 3 mice; 5A, n = 68 from 2 mice; and 4A5A, n = 33 from 1 mouse) were analyzed by SGS assay, and the raw data are shown.
(TIF)

Figure S8 **The extent of mutation in each amplicon of viral** ***env*** **in plasma.** The numbers of total mutations (*top*, gray), G-to-A mutations (*upper middle*, black), GA-to-AA mutations (*lower middle*, red), and GG-to-AG mutations (*bottom*, blue) within each amplicon are respectively shown.
(TIF)

Table S1 **Putative drug-resistance mutations potentially induced by APOBEC3D and APOBEC3F.** The table provides the drug-resistance mutation sites potentially induced by APOBEC3D and APOBEC3F.
(PDF)

Table S2 **Humanized mice used in this study.** A full list of the 82 humanized mice used in this study.
(PDF)
