# Supplementary figures and images for: APOBEC3D and APOBEC3F Potently Promote HIV-1 Diversification and Evolution in Humanized Mouse Model
Source: PLoS Pathog. 2014 Oct 16;10(10):e1004453. doi: 10.1371/journal.ppat.1004453 (PMC4199767; doi:10.1371/journal.ppat.1004453)

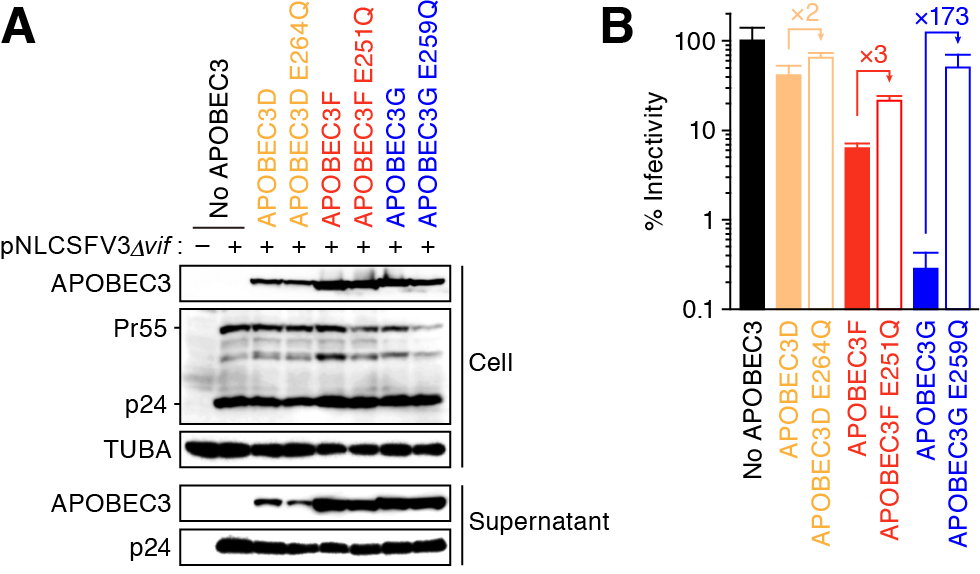

Supplement: Figure S1 — Anti-viral activity of WT and mutated APOBEC3 proteins in vitro . Two micrograms of pNLCSFV3Δvif was cotransfected with 100 ng of flag-tagged APOBEC3 expression plasmid into 293T cells. (A) Western blotting. The input of cell lysate was standardized to α-Tubulin (TUBA), and representative results are shown. (B) TZM-bl assay. The infectivity of released virus was determined by using TZM-bl cells. The infectivity of each virus is normalized to the value of no APOBEC3. The assay was performed in triplicate. The data represents average with SD. (TIF) [file ppat.1004453.s001.tif]

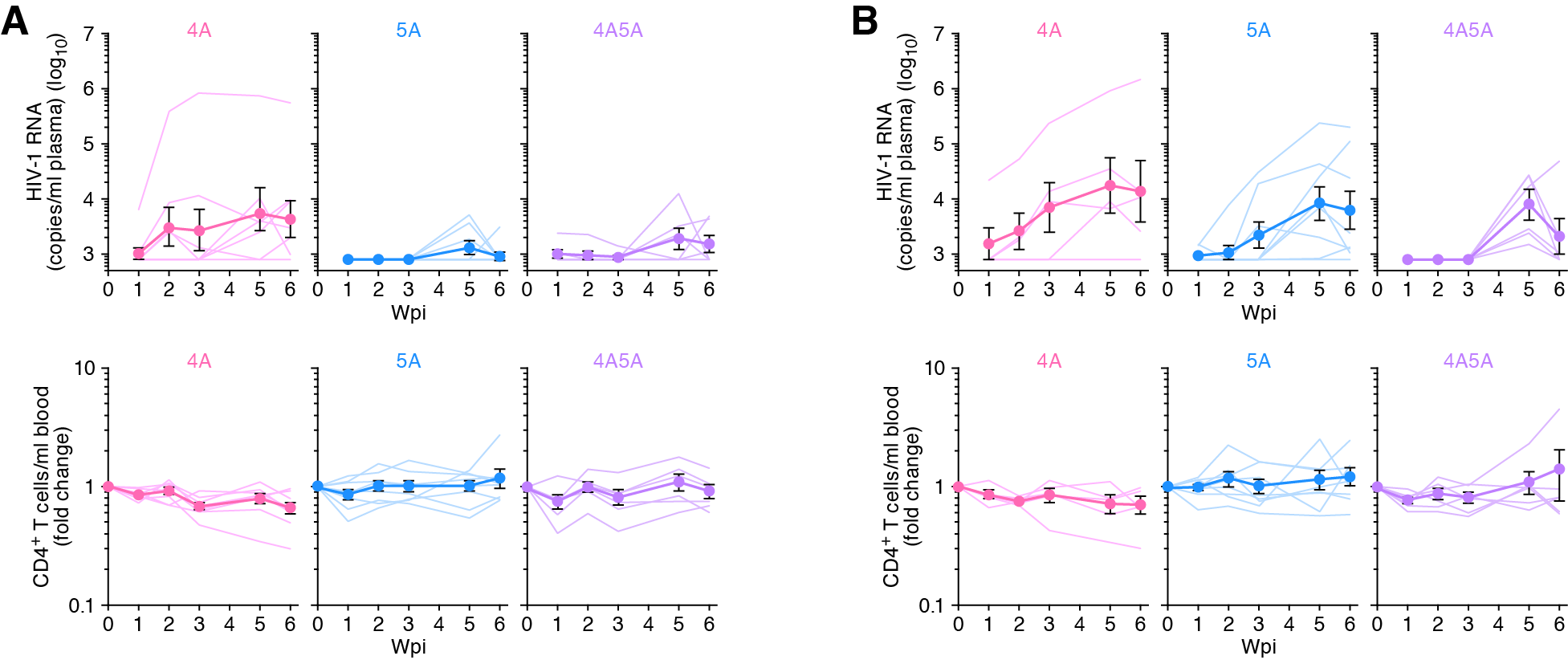

Supplement: Figure S2 — Dynamics of WT HIV-1 and HIV-1 vif mutants infection in humanized mice at higher doses. (A and B) Virus solutions containing 50 ng (A; 4A HIV-1 [n = 8], 5A HIV-1 [n = 8], and 4A5A HIV-1 [n = 6]) or 500 ng (B; 4A HIV-1 [n = 5], 5A HIV-1 [n = 8], and 4A5A HIV-1 [n = 6]) p24 antigens were intraperitoneally inoculated into humanized mice. The amount of viral RNA in plasma (top) and the level of peripheral CD4+ T cells (CD45+ CD3+ CD4+ cells) (bottom) were analyzed at 0, 1, 2, 3, 5, and 6 wpi. The averages are shown in circles with SEMs, and the values from each mouse are shown by line. In panel A, the detection limit of HIV-1 RNA is 800 copies/ml plasma. (TIF) [file ppat.1004453.s002.tif]

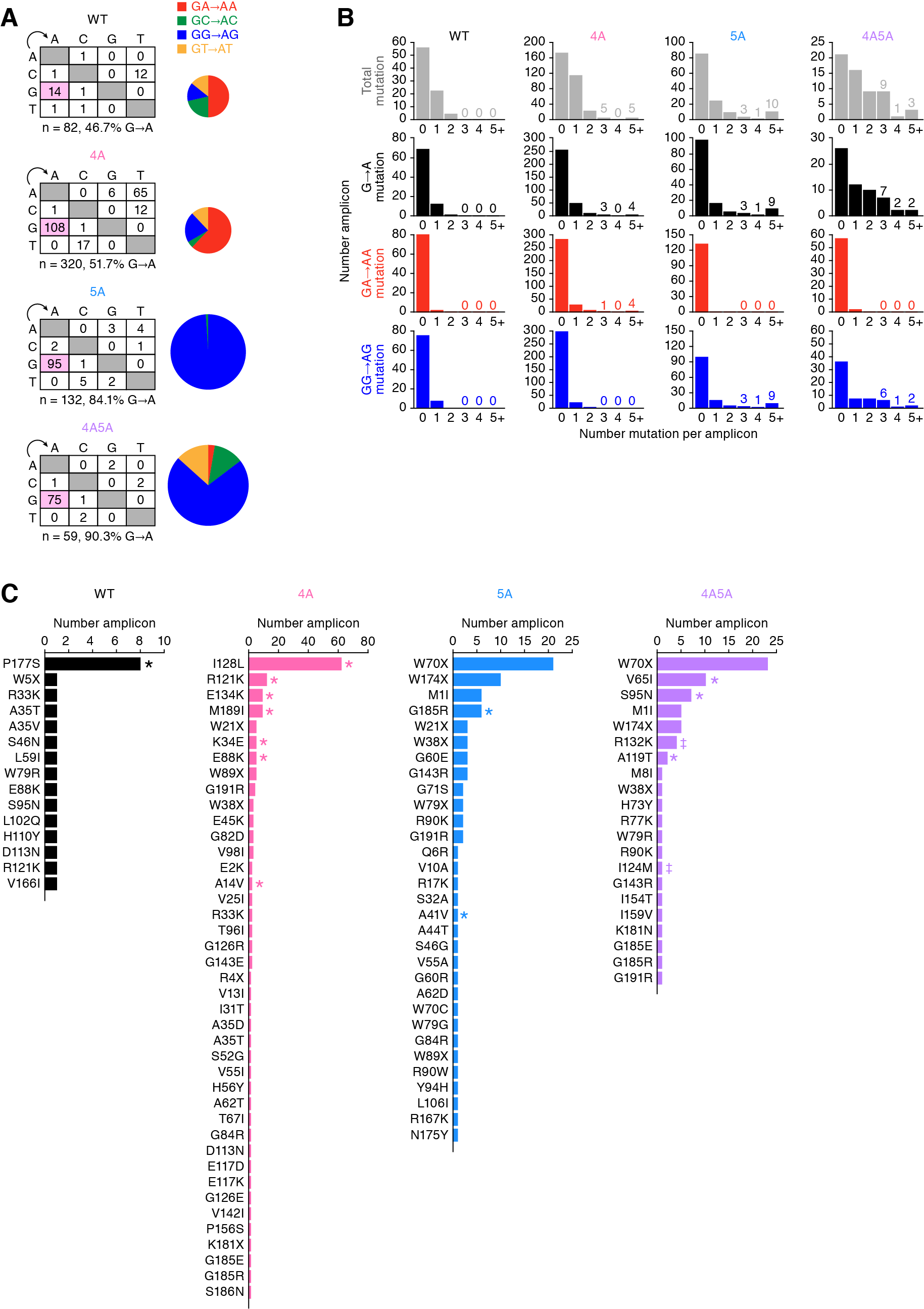

Supplement: Figure S3 — Mutations in vif ORF. The vif ORFs (5041–5619, 579 bases) of viral RNA in the spleen of infected mice (WT, n = 82 from 3 mice; 4A, n = 320 from 7 mice; 5A, n = 132 from 3 mice; and 4A5A, n = 59 from 1 mouse) were sequenced. (A) The mutation matrix (left) and the pie chart of G-to-A mutation (right) are shown. In the right panel, the diameters of pie charts represent the percentage of G-to-A mutations in total mutations. (B) The extent of mutation in each amplicon of vif ORF sequences. The numbers of total mutations (top, gray), G-to-A mutations (upper middle, black), GA-to-AA mutations (lower middle, red), and GG-to-AG mutations (bottom, blue) within each amplicon are respectively shown. (C) The vif amplicons harboring nonsynonymous mutations are summarized. “X” means stop codon mutation. The vif ORFs indicated by asterisks were used for the functional assay, and the results are shown in Figures 2E and 2F. In the panel of 4A5A, the amplicon harboring both R132K and I124M mutations (indicated by double daggers) were frequently detected and were used for the functional assay (Figures 2E and 2F). (TIF) [file ppat.1004453.s003.tif]

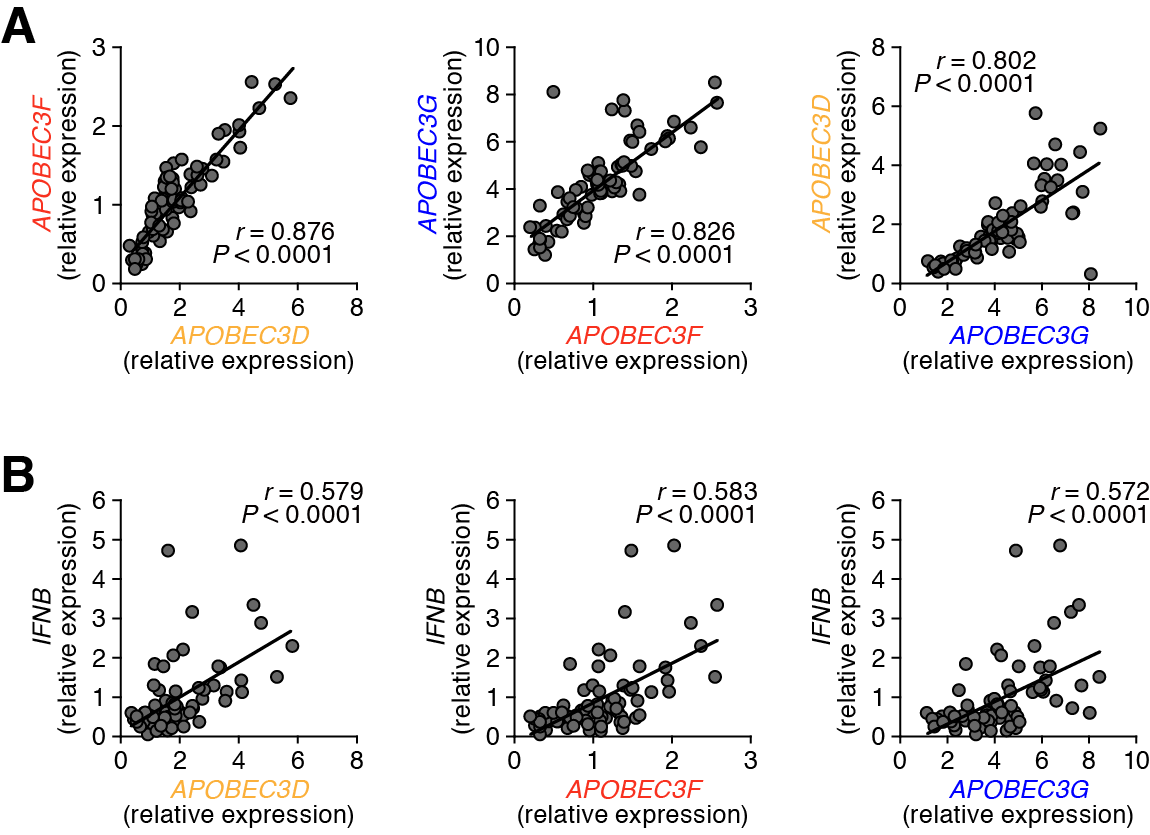

Supplement: Figure S4 — Correlation of APOBEC3 and IFNB expressions. The mRNA expression levels of APOBEC3D, APOBEEC3F, APOBEC3G, and IFNB in the splenic human CD4+ T cells of humanize mice (n = 73) were measured by real-time RT-PCR. The expression level of each gene was normalized to that of GAPDH and was shown as relative expression. The correlation between each APOBEC3 (A) and between APOBEC3 (x-axes) and IFNB (y-axes) (B) are respectively shown. The lines represent exponential approximation. Pearson correlation coefficient (r) was adopted to determine statistically significant correlation between each value. (TIF) [file ppat.1004453.s004.tif]

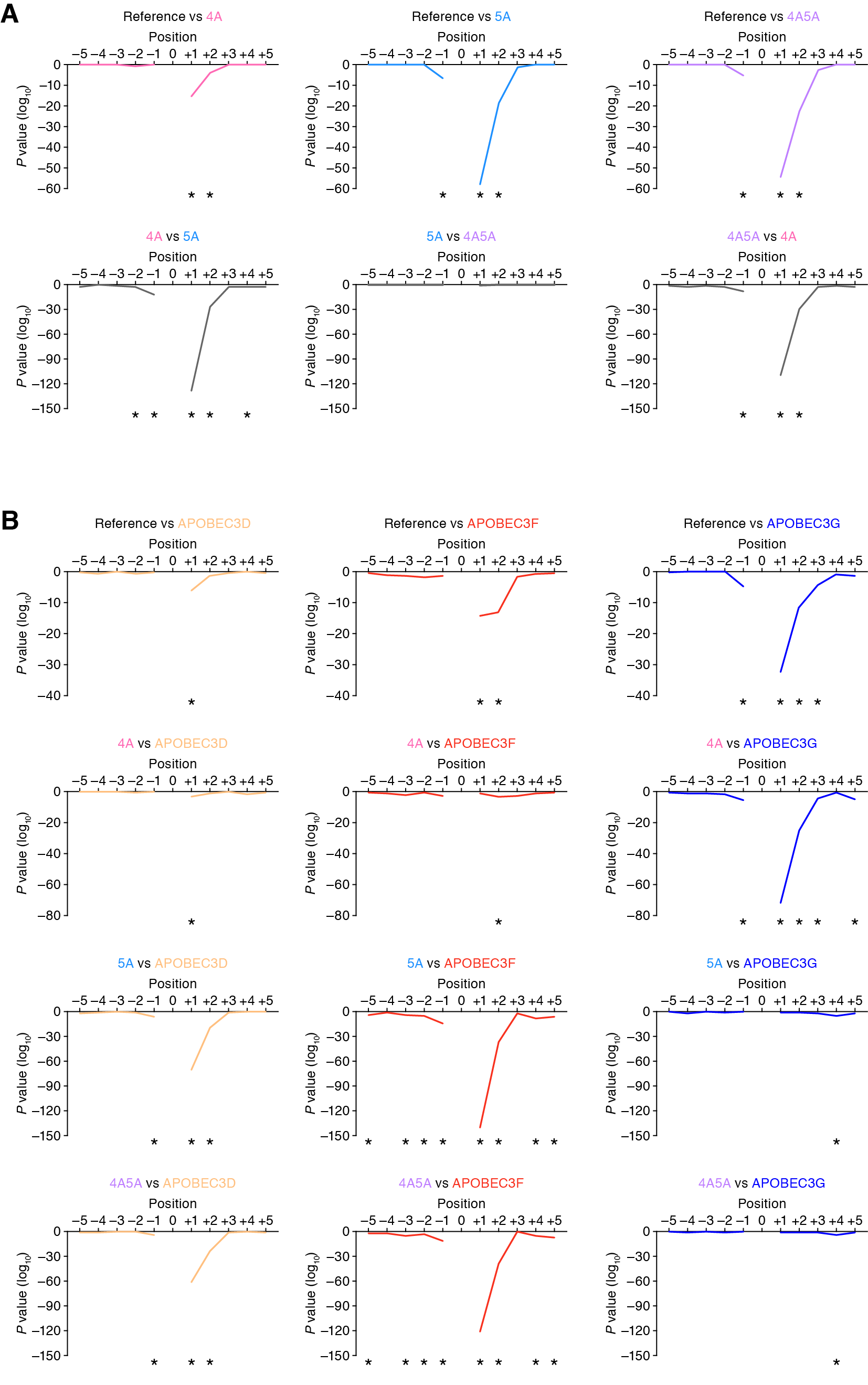

Supplement: Figure S5 — Statistical analyses on the preferential G-to-A mutation sites. The detected G-to-A mutation sites in the proviral DNA of vif-mutated HIV-1-infected mice (A) and in vitro experiments (B) were classified according to the nucleotides positioned between −5 to +5 from the detected G-to-A mutation sites (position 0; see also Figures 4F and 4G). Statistical differences at each position were determined by Chi-square test for independence, and the P values at each position is shown in y-axes. Statistically significant differences (P<0.001) are shown with asterisks. (TIF) [file ppat.1004453.s005.tif]

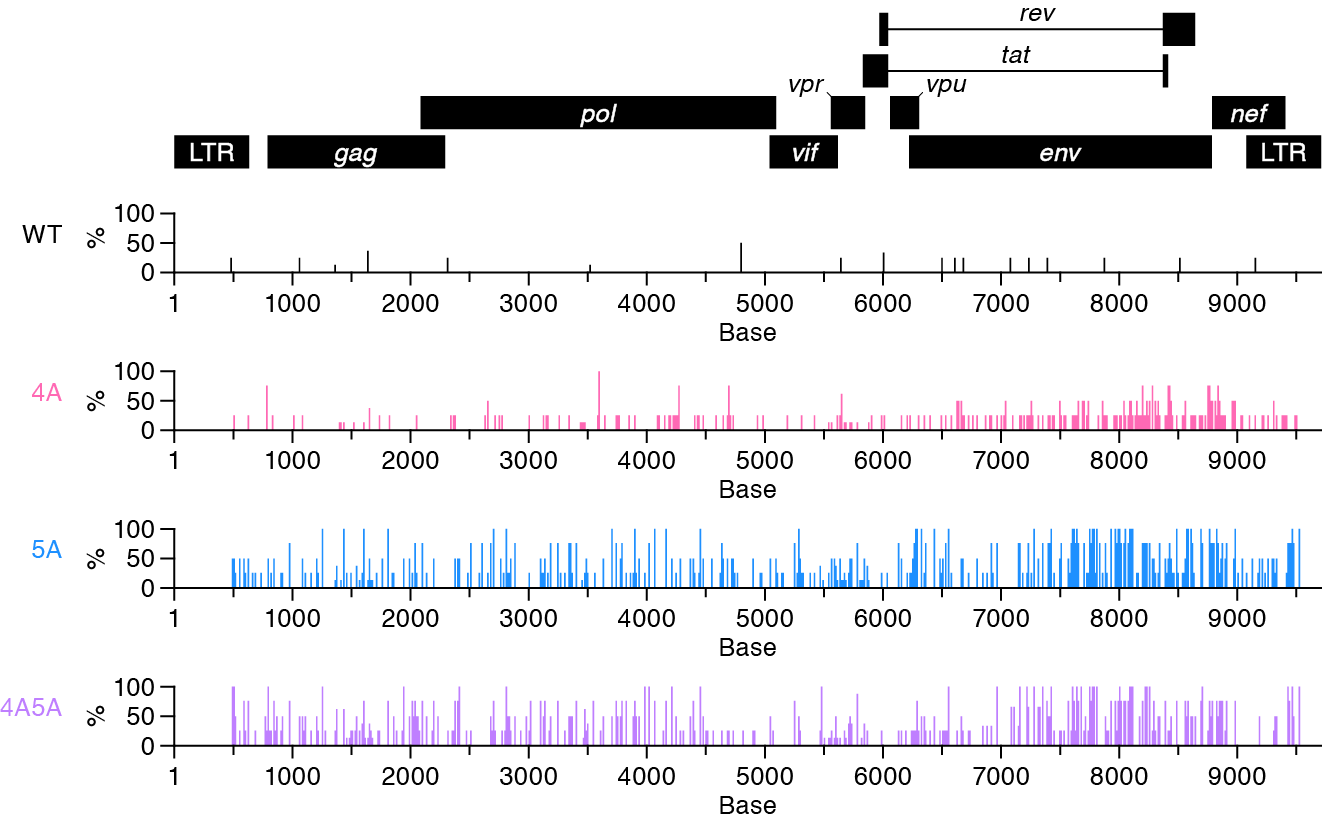

Supplement: Figure S6 — Mutations in the proviral DNA of infected humanized mice. The percentages of mutations in each site are summarized. (TIF) [file ppat.1004453.s006.tif]

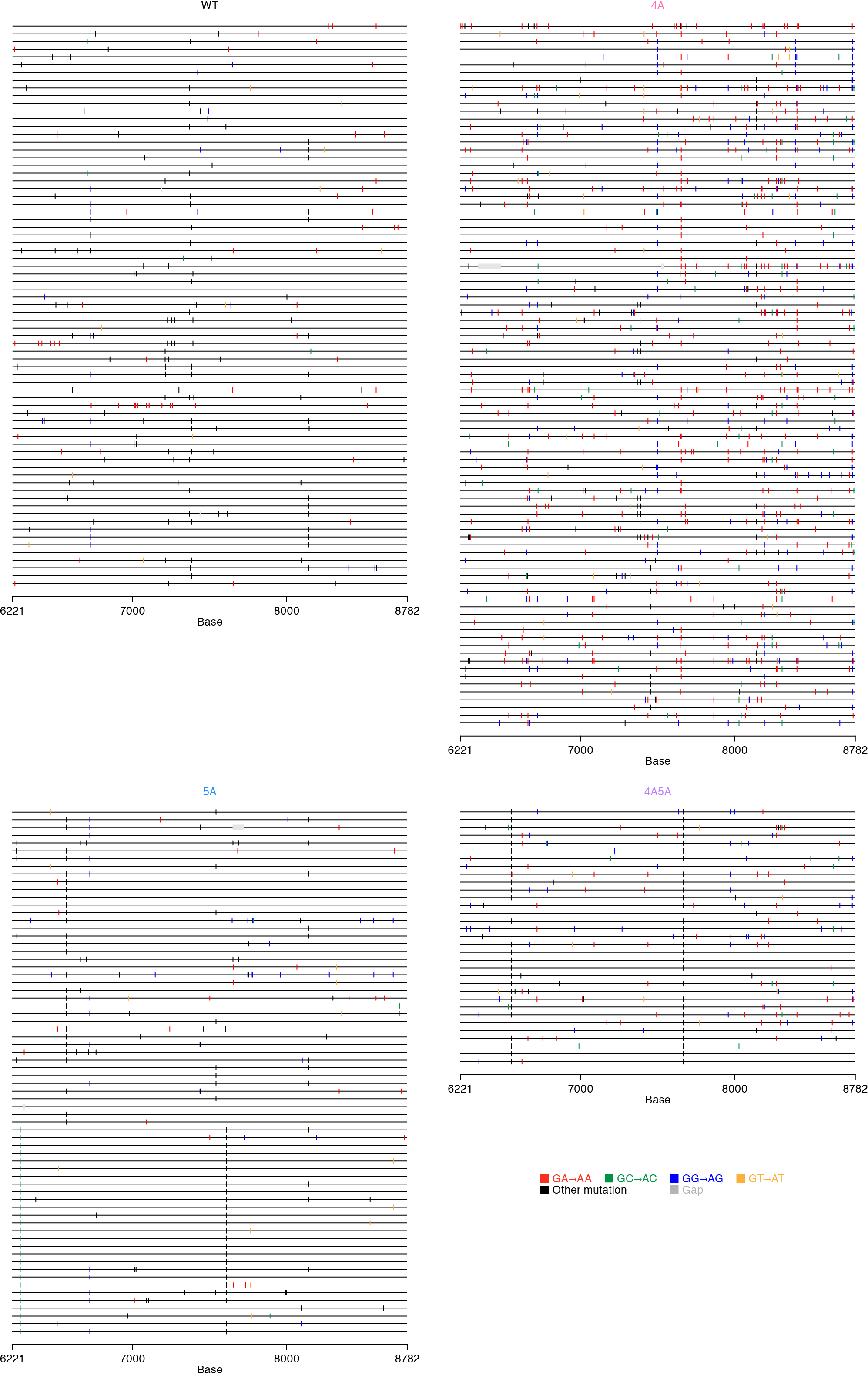

Supplement: Figure S7 — Raw data of SGS assay. The env ORF (6221–8782, 2,562 bases) of viral RNA in the plasma of infected mice (WT, n = 73 from 2 mice; 4A, n = 91 from 3 mice; 5A, n = 68 from 2 mice; and 4A5A, n = 33 from 1 mouse) were analyzed by SGS assay, and the raw data are shown. (TIF) [file ppat.1004453.s007.tif]

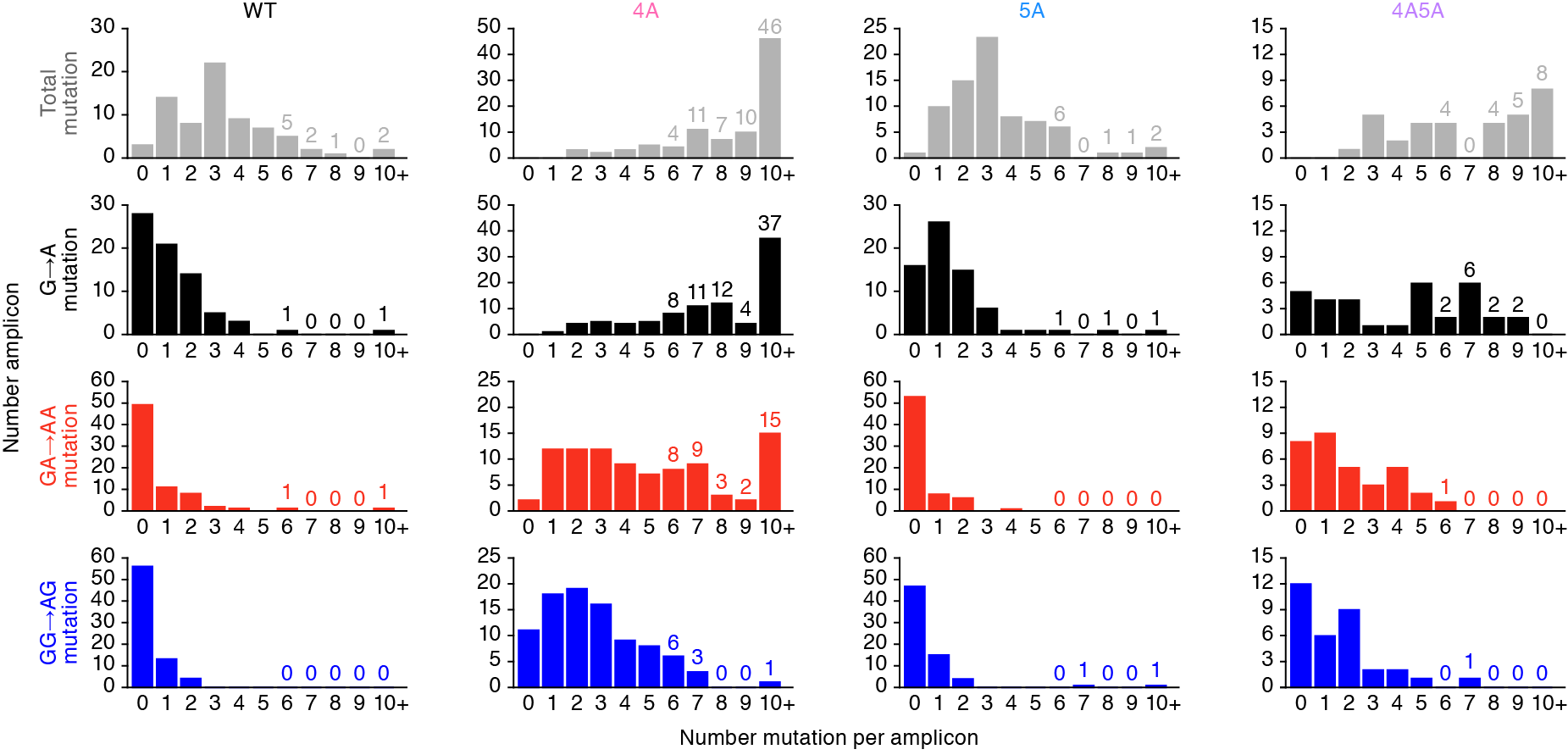

Supplement: Figure S8 — The extent of mutation in each amplicon of viral env in plasma. The numbers of total mutations (top, gray), G-to-A mutations (upper middle, black), GA-to-AA mutations (lower middle, red), and GG-to-AG mutations (bottom, blue) within each amplicon are respectively shown. (TIF) [file ppat.1004453.s008.tif]
