# Supplementary material for: APOBEC3D and APOBEC3F Potently Promote HIV-1 Diversification and Evolution in Humanized Mouse Model
Source: PLoS Pathog. 2014 Oct 16;10(10):e1004453. doi: 10.1371/journal.ppat.1004453 (PMC4199767; doi:10.1371/journal.ppat.1004453)
Supplement: Table S1 — Putative drug-resistance mutations potentially induced by APOBEC3D and APOBEC3F. The table provides the drug-resistance mutation sites potentially induced by APOBEC3D and APOBEC3F. (PDF) [file ppat.1004453.s009.pdf]

Table S1. Drug-resistance mutations potentially induced by APOBEC3D and APOBEC3F

Drug	Class*	Mutation	**Targeted motif**
Nelfinavir	PI	D30N	**GA-to-AA**
Indinavir	PI	M46I	**GA-to-AA**
Stavudine	NRTI	D67N	**GA-to-AA**
Zidobudine	NRTI	D67N	**GA-to-AA**
Rilpivirine	NNRTI	E138K	**GA-to-AA**

*PI, protease inhibitor; NRTI, nucleoside reverse transcriptase inhibitor; NNRTI, non-nucleoside reverse transcriptase inhibitor.
