# Supplementary material for: APOBEC3D and APOBEC3F Potently Promote HIV-1 Diversification and Evolution in Humanized Mouse Model
Source: PLoS Pathog. 2014 Oct 16;10(10):e1004453. doi: 10.1371/journal.ppat.1004453 (PMC4199767; doi:10.1371/journal.ppat.1004453)
Supplement: Table S2 — Humanized mice used in this study. A full list of the 82 humanized mice used in this study. (PDF) [file ppat.1004453.s010.pdf]

Table S2. Humanized mice used in this study (1 of 2 pages)

Mouse no.	Recipient mouse		Transplanted hHSCs§		Inoculated virus	Dose (ng p24)	Inoculated age (weeks)
	Lot no.*	Sex†	Donor lot‡	No. of cells			
1	135	F	A	150,000	4A5A	5	18
2	135	F	A	150,000	WT	5	18
3	135	F	A	150,000	WT	5	18
4	135	M	A	150,000	4A	5	18
5	135	M	A	150,000	4A	5	18
6	135	M	A	150,000	4A	5	18
7	136	F	B	170,000	5A	5	17
8	136	F	B	170,000	4A5A	5	17
9	136	F	B	170,000	5A	5	17
10	136	F	B	170,000	4A5A	5	17
11	136	M	B	170,000	5A	5	17
12	136	M	B	170,000	WT	5	17
13	136	M	B	170,000	Mock	5	17
14	136	M	B	170,000	Mock	5	17
15	136	M	B	170,000	Mock	5	17
16	138	M	C	170,000	4A	5	17
17	141	F	C	110,000	4A	5	15
18	141	F	C	110,000	5A	5	15
19	141	F	C	110,000	5A	5	15
20	141	M	C	110,000	4A	5	15
21	141	M	C	110,000	5A	5	15
22	141	M	C	110,000	5A	5	15
23	146	F	C	140,000	4A	5	19
24	146	F	C	140,000	4A	5	19
25	146	M	C	140,000	5A	5	19
26	146	M	C	140,000	4A5A	5	19
27	148	F	A	130,000	5A	5	19
28	148	F	A	130,000	5A	5	19
29	148	F	A	130,000	4A5A	5	19
30	148	M	A	130,000	4A5A	5	19
31	148	M	A	130,000	4A	5	19
32	149	F	C	140,000	4A	5	18
33	149	F	C	140,000	4A	5	18
34	149	F	C	140,000	5A	5	18
35	149	F	C	140,000	5A	5	18
36	149	M	C	140,000	4A5A	5	18
37	149	M	C	140,000	4A5A	5	18
38	150	F	C	80,000	4A	500	18
39	150	F	C	80,000	4A	500	18
40	150	F	C	80,000	5A	500	18
41	150	F	C	80,000	5A	500	18
42	150	F	C	80,000	4A5A	500	18

(Continued on following page)

Table S2. Humanized mice used in this study (2 of 2 pages)

Mouse no.	Recipient mouse		Transplanted hHSCs§		Inoculated virus	Dose (ng p24)	Inoculated age (weeks)
	Lot no.*	Sex†	Donor lot‡	No. of cells			
43	150	F	C	80,000	4A5A	500	18
44	150	M	C	80,000	4A	500	18
45	150	M	C	80,000	5A	500	18
46	150	M	C	80,000	4A5A	500	18
47	151	F	C	120,000	4A	500	17
48	151	F	C	120,000	4A	50	17
49	151	M	C	120,000	5A	500	17
50	151	M	C	120,000	4A5A	500	17
51	152	F	C	160,000	4A	50	15
52	152	M	C	160,000	5A	50	15
53	152	M	C	160,000	5A	50	15
54	152	M	C	160,000	4A5A	50	15
55	152	M	C	160,000	4A5A	50	15
56	155	F	C	100,000	4A	50	15
57	155	F	C	100,000	4A	50	15
58	155	F	C	100,000	4A	50	15
59	155	F	C	100,000	5A	50	15
60	155	F	C	100,000	4A5A	50	15
61	156	F	C	100,000	WT	5	16
62	156	M	C	100,000	WT	5	16
63	157	F	C	110,000	WT	5	14
64	157	M	C	110,000	WT	5	14
65	165	F	A	150,000	4A5A	50	24
66	165	M	A	150,000	4A	50	24
67	165	M	A	150,000	4A	50	24
68	166	F	B	140,000	5A	50	23
69	166	F	B	140,000	4A5A	50	23
70	166	F	B	140,000	5A	50	23
71	166	F	B	140,000	4A5A	50	23
72	166	M	B	140,000	5A	50	23
73	171	F	C	110,000	4A	50	21
74	171	F	C	110,000	5A	50	21
75	171	M	C	110,000	5A	50	21
76	175	F	A	150,000	4A5A	500	30
77	175	M	A	150,000	4A	500	30
78	176	F	B	140,000	5A	500	29
79	176	F	B	140,000	4A5A	500	29
80	176	M	B	140,000	5A	500	29
81	181	F	B	140,000	5A	500	27
82	181	M	B	140,000	5A	500	27

* Nineteen lots of newborn NOG mice were used for the recipient.

† F, female; M, male.

‡ NOG-hCD34 mice were reconstructed with one of 3 donors.

§ hHSCs, human $CD34^+$ hematopoietic stem cells.
